# RegulatorTrail: a web service for the identification of key transcriptional regulators

**DOI:** 10.1093/nar/gkx350

**Published:** 2017-05-02

**Authors:** Tim Kehl, Lara Schneider, Florian Schmidt, Daniel Stöckel, Nico Gerstner, Christina Backes, Eckart Meese, Andreas Keller, Marcel H. Schulz, Hans-Peter Lenhof

**Affiliations:** 1 Center for Bioinformatics, Saarland Informatics Campus, Saarland University, 66123 Saarbrücken, Germany; 2 Cluster of Excellence Multimodal Computing and Interaction, Saarland Informatics Campus, 66123 Saarland University, Saarbrücken, Germany; 3 Max Planck Institute for Informatics, Saarland Informatics Campus, 66123 Saarbrücken, Germany; 4 Human Genetics, Saarland University, 66421 Homburg, Germany

## Abstract

Transcriptional regulators such as transcription factors and chromatin modifiers play a central role in most biological processes. Alterations in their activities have been observed in many diseases, e.g. cancer. Hence, it is of utmost importance to evaluate and assess the effects of transcriptional regulators on natural and pathogenic processes. Here, we present RegulatorTrail, a web service that provides rich functionality for the identification and prioritization of key transcriptional regulators that have a strong impact on, e.g. pathological processes. RegulatorTrail offers eight methods that use regulator binding information in combination with transcriptomic or epigenomic data to infer the most influential regulators. Our web service not only provides an intuitive web interface, but also a well-documented RESTful API that allows for a straightforward integration into third-party workflows. The presented case studies highlight the capabilities of our web service and demonstrate its potential for the identification of influential regulators: we successfully identified regulators that might explain the increased malignancy in metastatic melanoma compared to primary tumors, as well as important regulators in macrophages. RegulatorTrail is freely accessible at: https://regulatortrail.bioinf.uni-sb.de/.

## INTRODUCTION

Transcriptional regulators like transcription factors (TFs), coregulators and chromatin modifiers are proteins that control the expression of genes by promoting or inhibiting their transcription and that are involved in the regulation of most biological processes and signaling pathways (1). Mutations in transcriptional regulators or regulatory regions can lead to alterations of transcriptional programs (2). Hence, such mutations can cause diseases (2,3). For instance, mutations in several hepatocyte nuclear factors (HNFs) (4) and in the insulin promoting factor PDX1 (5) are associated with diabetes. Many transcriptional regulators have also been described in the context of tumor progression and metastasis (6), e.g. several members of the NF-κB family (7–9). Many regulators are even described as (proto-)oncogenes or tumor suppressor genes (10). The most prominent example is the tumor suppressor gene *TP53*, for which alterations in a variety of cancer types have been described (11). Their capability to control the transcription of a large number of genes makes transcriptional regulators interesting candidates as putative drug targets in cancer therapy (12–14).

Due to their inherent importance, it is crucial to identify transcriptional regulators that might explain expression changes between two groups of samples, e.g. disease versus control. In the following, we present a non-exhaustive list of algorithms that have been proposed for this purpose. We start our discussion with methods that use a predefined collection of regulator–target interactions (RTIs). Here, a pair (regulator, target gene) is defined as an RTI, if a binding of the regulator to a regulatory region (promotor, enhancer, etc.) of the target gene has been experimentally determined.

A first group of approaches was designed to find individual regulators whose target genes have a significant overlap with a list of differentially expressed genes (15,16).

A second group of approaches, discussed in this section, identifies important regulators based on gene expression data. For example, the ‘regulatory impact factors’ *RIF1* and *RIF2* (17) measure the degree of differential co-expression between a regulator and all its target genes. A further approach that requires gene expression data is the so-called *Correlation Set Analysis* (18), a method that unveils essential regulators in disease populations by calculating the mean correlation of all target pairs. We recently developed an enrichment-based method called REGGAE that prioritizes regulators based on correlation coefficients from gene expression data (Kehl *et al.*, in submission). A graph-based method for the identification of key regulators in a regulatory network has been developed by Gonçalves *et al.* (19). A *t*-test-based approach, called *wPGSA*, that utilizes the probability of regulation in replicated ChIP-Seq experiments was presented by Kawakami *et al.* (20). Poos *et al.* published a machine learning approach, called *MIPRIP* (21), that predicts the most influential regulators for a single target gene. Gonçalves *et al.* presented *Regulatory Snapshots*, a web server for the identification of important regulatory modules (22) using time series gene expression data.

Another group of methods is based on genome-wide TF binding predictions. Exclusively sequence-based prediction methods, which screen the genome using position weight matrices, usually generate many false positive predictions. Recent studies verified that the number of false positive predictions can be substantially reduced by combining epigenetics data with sequence-based TF binding predictions (23,24). Several methods incorporating epigenetics data have been proposed, e.g. *CENTIPEDE* (23), *PIQ* (25), *MILLIPEDE* (26), *BinDNase* (27), *HINT-BC* (28) or *TEPIC* (29). These predictions can be used in downstream applications, e.g. the *PASTAA* web service calculates TF binding affinities based on sequence specificity and applies the hypergeometric test to infer co-regulated target genes (30). TF binding predictions can also be used as features to build interpretable, predictive models of gene expression (29,31–34). An overview of the essential features of all methods discussed above is provided in Supplementary Table S1.

Here, we present RegulatorTrail, a new web service that provides rich functionality for the identification of key transcriptional regulators. In contrast to existing web servers that are specifically tailored to a single application scenario, we designed RegulatorTrail as a general framework offering eight distinct methods to identify key transcriptional regulators. Moreover, we ensured that RegulatorTrail offers at least one method from the different methodological classes sketched above and hence provides solutions for four specific application scenarios. Besides the wide range of algorithms, RegulatorTrail also provides comprehensive collections of RTIs and position-specific energy matrices (PSEMs) extracted from several databases (cf. ‘Resources and supported file formats’ section). In order to find commonly regulated biological processes or signaling pathways, the respective results can be further processed in a downstream enrichment analysis using the GeneTrail2 enrichment pipeline (35). This versatility combined with its intuitive web interface and the well-documented RESTful API set RegulatorTrail apart from other approaches. We demonstrate the capabilities of our web server based on two case studies. First, we analyze mRNA microarrays from melanoma patients (NCBI GEO (36): GSE7553 (37)) to find transcriptional regulators that might be responsible for expression differences between metastatic and non-metastatic tumors. Second, we perform an integrative analysis of open-chromatin regions and corresponding gene expression estimates of macrophage data (BLUEPRINT (38) sample ID: S001S7) to infer potentially important transcriptional regulators.

## WORKFLOW

**Figure 1.**
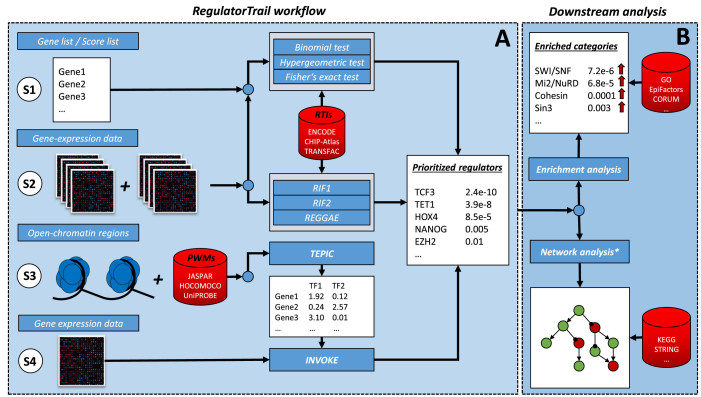
General overview of the RegulatorTrail workflow. S1–S4 represent four different application scenarios. In each scenario, different types of input files are required to identify influential regulators. The resulting regulator list can then be further investigated using the functionality of GeneTrail2 (downstream analysis). *Network analysis can only be applied in Scenarios 1 and 2.

RegulatorTrail provides a variety of methods for the identification of important transcriptional regulators that can be applied to four distinct application scenarios. An overview of the different workflows is presented in Figure 1A. In each scenario, different input data is required for the computation of the most influential regulators (cf. ‘Resources and supported file formats’ section). The different approaches utilize our comprehensive collections of RTIs and PSEMs. In all scenarios, the output is a prioritized (sorted) list of transcriptional regulators or regulated target genes respectively that can be visualized in the web browser or downloaded in a variety of standard file formats, including CSV, JSON, Excel and PDF. Additionally, the resulting lists can be further analyzed with the enrichment or network analysis functionality of GeneTrail2 (35) (cf. Figure 1B). Expected runtimes for all algorithms and different inputs can be found in Supplementary Table S2.

**Scenario 1:** in the first scenario, a user can upload a list of differentially expressed genes, e.g. genes that are differentially expressed between two groups of samples. Then the user can choose a collection of RTIs from our web server. Based on the gene list and the selected RTIs, RegulatorTrail identifies transcriptional regulators, whose set of target genes have a significant overlap with the uploaded gene list. For this purpose, three statistical tests are offered: a binomial test as described by Yang *et al.* (16), a hypergeometric test as presented by Essaghir *et al.* (15) and the Fisher's exact test. For *P*-value adjustment, RegulatorTail offers eight methods (cf. Supplementary Table S3), e.g. the false discovery rate (FDR)-adjustment method presented by Benjamini and Yekutieli (39). Finally, RegulatorTrail outputs a list of regulators sorted with respect to the adjusted *P*-values. For gene lists of size 250, the average runtime for the hypergeometric test and the Fisher's exact test is 25 s and for the binomial test 4 min. Essaghir *et al.* considered such a scenario to find potential biomarkers common to multiple cancer types (40).

**Scenario 2:** in the second scenario, the user can upload a matrix that contains normalized gene expression values, where the samples belong to two groups of interest, e.g. disease and control. In a first step, expression differences between the two groups can be calculated. To this end, we provide a variety of methods. Among them standard measures like fold change, z-score and signal-to-noise ratio, as well as dependent and independent versions of widely used statistical tests like *t*-test and Wilcoxon rank-sum test. For count data, we additionally integrated the *DESeq2* (41), *edgeR* (42) and *RUVSeq* (43) R-packages. In a second step, the user selects lists of up- or downregulated genes. The respective lists can then be used to identify regulators with over-represented target gene sets as described in the first scenario. For the second scenario, RegulatorTrail provides three further approaches that utilize expression correlations between regulators and targets to prioritize the considered regulators: *RIF1, RIF2* (17) and *REGGAE*. Besides the sorted regulator lists, these methods additionally provide information on whether the regulator has an activating or repressing effect. For a gene expression matrix with around 13 000 protein coding genes, 38 samples per group and a filtered gene list of size 250, the average runtime of this scenario is around 10 s for the regulatory impact factors and ∼3 min for a *REGGAE* analysis. Yao *et al.* considered such a scenario to identify genes associated with renal cell carcinoma (44).

**Scenario 3:** in the third scenario, the user can upload a BED file containing candidate regions for TF binding, which can be derived from open-chromatin data, e.g. DNase-hypersensitive sites (DHS) and TF-footprints, as well as from histone modification ChIP-seq data, e.g. H3K4me3 peaks. From the provided set of candidate regions, RegulatorTrail extracts those that overlap with windows of user-defined size that are centered at the most 5΄ transcriptional start site of all genes. Using the *TEPIC* framework (29), gene-TF binding scores are computed for all genes and a species-specific set of distinct TFs using an exponential decay formulation (45). The resulting gene-TF scores are provided as a tab-separated matrix that can either be used in a downstream enrichment analysis or to build a predictive model of gene expression (cf. Scenario 4). For genome-wide analysis of TF binding affinities, the average runtime is around 8 min using the entire collection of PSEMs. A similar scenario has already been considered in (46).

**Scenario 4:** in addition to the BED file required in Scenario 3, also gene expression data must be uploaded to be able to perform an *INVOKE* (identification of key regulators) analysis. INVOKE follows a two-step approach. First, gene-TF binding scores are computed as described in the third scenario. Second, these scores are used as features in a linear regression model with either lasso, ridge or elastic net penalty to predict gene expression. Training and evaluating the model leads to three different outputs: model performance is assessed by calculating Pearson correlation, Spearman correlation and the mean-squared error (MSE) between predicted and measured gene expression on test data. Furthermore, we report a list of features with non-zero regression coefficients. These features were selected during model training, thus the corresponding TFs are likely to play an essential role in transcriptional regulation of the analyzed sample. In addition, a bar plot showing the top features, ranked according to their regression coefficients, is provided. Using lasso regularization, the expected runtime of this scenario is around 4 min. If additionally, the performance of the model should be calculated, the average runtime increases to ∼7 min. This scenario has already been applied in (29). Similar approaches have also been pursued in (31–34).

## RESOURCES AND SUPPORTED FILE FORMATS

Currently, RegulatorTrail enables users to analyze regulatory interactions for five different organisms: *Homo sapiens, Mus musculus, Rattus norvegicus, Drosophila melanogaster* and *Caenorhabditis elegans*. Our web service accepts various input file formats through which the user can provide gene lists, gene expression data or genomic regions. Gene lists or gene expression data must be provided as tab-separated text files, where each line contains a single gene followed by associated gene expression measurements. Additionally, the integrated *GSE* file parser can be used to download microarray experiments from the *NCBI Gene Expression Omnibus* (GEO) (36). In both cases, RegulatorTrail automatically detects and normalizes the used identifiers based on mapping information from *UniProt* (47) and *NCBI* (48). Genomic regions must be provided in standard BED format.

The different algorithms offered by RegulatorTrail rely on third-party resources that contain information on TF binding motifs or interactions between regulators and associated target genes.

All approaches offered for Scenario 1 and 2 require information on RTIs. To this end, we have built a comprehensive collection of RTIs based on seven databases: *ChEA* (49), *ChIP-Atlas* (chip-atlas.org), *ChipBase* (50), *ENCODE* (51), *JASPAR* (52), *SignaLink* (53) and *TRANSFAC* (54). However, the included databases provide different levels of information on regulators and their putative target genes: (i) predefined RTIs extracted from e.g. literature, (ii) binding sites of regulators extracted from e.g. ChIP-Seq experiments and (iii) RTIs determined by assigning regulator binding sites to neighbored target genes based on their distances to the transcription start site (TSS) of the genes. More precisely, a regulator is assigned to a gene if the binding site is in an interval around the TSS. The different databases provide different RT assignments based on symmetric or asymmetric intervals around the TSS: [−1 kb, +1 kb], [−5 kb, +5 kb], [−10 kb, +10 kb], [−10 kb, +1 kb]. For consistency reasons, we processed the available information on binding sites for all databases such that all four proposed interval assignments can be selected by the user. Users can also select which RTI databases should be used for their analysis and they can even upload their own set of RTIs.

In Scenarios 3 and 4, PSEMs that are derived from position count matrices (PCMs) are used. We downloaded the PCMs from several databases: *TRANSFAC* (54), *HOCOMOCO* (55), *JASPAR* (52) and the *Kellis lab ENCODE Motif database* (56). To exclude PCMs of low quality, we calculate the information content (IC) of each PCM and remove all matrices from our collection that have an IC value above a threshold. If the databases contain multiple PCMs for the same TF, only the most informative PCM is considered. In case that a TF has a known secondary binding motif, we also keep the alternative PCM in our collection. Finally, we have converted all PCMs to PSEMs according to a mismatch energy formulation introduced by Berg and von Hippel (57).

For all scenarios, a reference genome and gene annotations are required, which were downloaded from *Ensembl* (58), *GENCODE* (59) and *UCS*C (60).

For all databases, we have implemented update routines that will regularly be used to create new database versions. Provenance data including retrieval dates of all databases as well as detailed descriptions of all processing steps are provided on the RegulatorTrail website.

## SOFTWARE ARCHITECTURE AND IMPLEMENTATION

**Figure 2.**
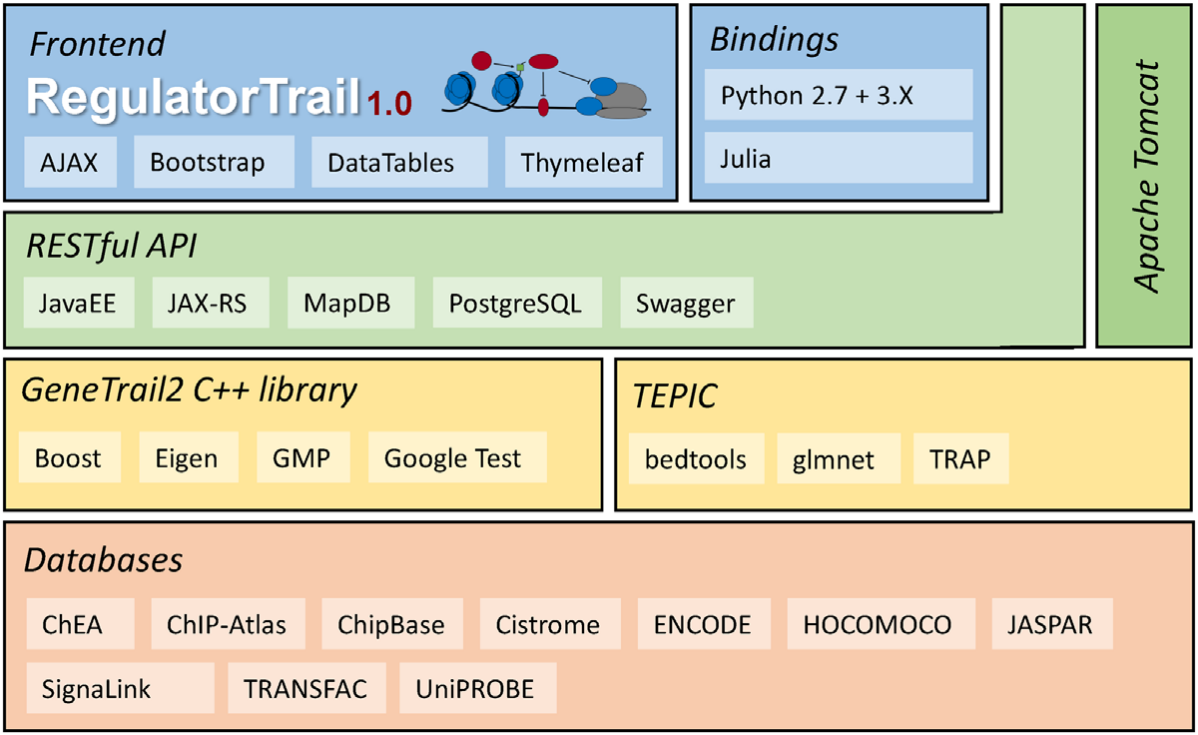
The different layers of the RegulatorTrail architecture. Core algorithms are provided by the *TEPIC* framework and the *GeneTrail2* C++ library. On top of this, we have built a RESTful API that manages the corresponding algorithms and provides an interface for our web frontend, as well as the Python and Julia bindings.

RegulatorTrail is based on the modular architecture of the *GeneTrail2* web service (35). This architecture can be represented by a layered hierarchy with distinct functional components as shown in Figure 2. The first component of the top layer is the web interface of RegulatorTrail that was implemented using the Thymeleaf template engine and the Bootstrap 3 web framework. This web interface interacts with the underlying web server via a JAX-RS based RESTful API, which provides interfaces to start an analysis or to query respective results. This API also allows users to incorporate RegulatorTrail into existing third-party pipelines. Additionally, we provide Python 2.7, Python 3 and Julia bindings that can directly be used to script our web service. The actual processing tasks are performed using the *TEPIC* framework (29) and the *GeneTrail2* (35) C++ library, which we extended with algorithms for regulator effect analysis.

## CASE STUDIES

Due to space constraints, we focused on two case studies illustrating the more elaborate application scenarios 2, 3 and 4 (cf. ‘Workflow’ section). In the first case study, we analyzed gene expression data of melanoma patients to identify regulators that might be responsible for the increased malignancy of cases with metastatic melanoma. In the second case study, we performed an integrative analysis of open-chromatin regions and gene expression data to find key regulators of macrophages.

### Comparison of metastatic and non-metastatic melanoma

Melanoma is one of the most severe types of skin cancer. Especially cases with metastatic melanoma have a poor prognosis with an average survival time of around 1 year (61).

We analyzed a microarray dataset provided by Riker *et al.* (37) (GSE7553) to find transcriptional regulators that have a significant impact on genes that are upregulated in metastatic compared to non-metastatic melanoma samples. First, we used RegulatorTrail's integrated GEO file parser to download and process the corresponding GSE file. In a second step, we selected metastatic and primary melanoma samples as case group and control group respectively. A shrinkage *t*-test (62) was used to compute expression differences between the two groups and to select upregulated genes. Finally, we performed a REGGAE analysis to identify important regulators. The parameters of the REGGAE analysis and corresponding results are provided in Supplementary Tables S4 and S5. The top 15 transcriptional regulators provided by REAGGE can be found in Table 1. Of these 15 regulators, 13 have already been described in the context of melanoma (e.g. ZBTB7A (63), MITF (64) and ATF2 (65,66)) and twelve are known to be involved in metastasis or tumor progression in melanoma (e.g. GATA3 (67)) or other cancer types (e.g. CEBPA (68)). Moreover, our analysis revealed a set of eleven regulators that show decreased activity in metastatic melanoma compared to primary tumors and among them four known tumor suppressor genes. In particular, downregulation of ZBTB7A or TP63 has already been associated with poor prognosis of melanoma patients. ZBTB7A is known to promote metastasis in melanoma (63) and TP63 is associated with resistance to therapeutic agents (69). Additionally, we identified six regulators that have an increased activity in patients with metastatic tumors and among them two that have already been described as oncogenes: MITF and ATF2. The former is known to be amplified in malignant melanoma (64) and the latter is associated with the progression of the disease and even investigated as potential drug target for the therapy of melanoma (65,66).

**Table 1.** Top 15 regulators provided by the REGGAE analysis of upregulated genes for the comparison of metastatic and non-metastatic melanoma patients

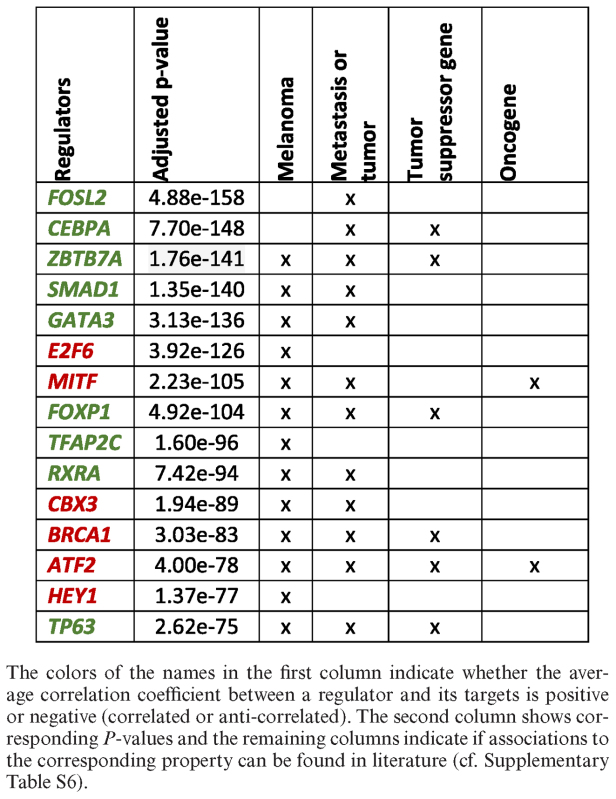

### Inferring key transcriptional regulators of macrophages

Macrophages are cells with diverse functions. They have phagocytic activity, play an essential role in the innate immune system, as well as in the adaptive immune system (70). Thus, understanding the regulatory mechanisms in macrophages is of general interest.

We analyzed DHS (S001S745.ERX616976) and gene expression data (S001S712) of macrophages extracted from venous blood (S001S7) in the scope of the BLUEPRINT epigenomics project (38). We uploaded the BED file containing the DHS regions as well as corresponding gene expression values to RegulatorTrail and selected GRCh38 as the reference genome. Next, we selected a window of 50 000 bp around the 5΄-TSS of genes to compute gene-TF binding scores. Using the INVOKE component of RegulatorTrail, we have trained a linear regression model with elastic net penalty and the following default parameters: a 6-fold outer cross-validation, a 6-fold inner cross-validation and an alpha step size of 0.1. In order to judge the quality of the learned model, RegulatorTrail computes three different performance measures on test data, comparing predicted and measured gene expression across the outer folds. The model achieved a Pearson correlation of 0.616, a Spearman correlation of 0.666 and an MSE of 0.623.

**Figure 3.**
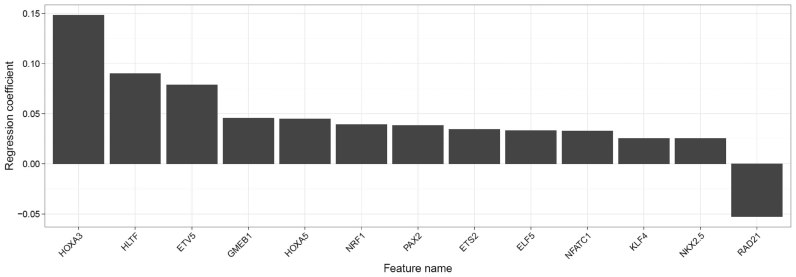
Bar plot showing the non-zero regression coefficients derived by an INVOKE analysis on macrophage data from BLUEPRINT. For visualization, we used an absolute value cut-off of 0.025.

In total, 13 TFs were selected with an absolute regression coefficient ≥0.025 and are shown in the bar plot in Figure 3. We found evidence that these 13 TFs are related to gene regulation in macrophages. Al Sadoun *et al.* have recently shown that the top ranked regulator, HOXA3, promotes macrophage maturation (71). Another factor, HLTF, is known to be targeted by the HIV-1 protein Vpr in T-cells and macrophages. As a consequence, HLTF is degraded, which negatively affects DNA repair mechanisms in infected cells (72). ETS2 is known to regulate macrophages during inflammation and to be involved in the regulation of tumor associated macrophages (73). The Kruppel Like Factor 4 (KLF4) is a zinc finger protein that can induce macrophage differentiation (74). Additionally, KLF4 was identified to regulate macrophage polarization (75). A list of all 13 TFs and references to literature describing the role of those TFs in macrophages are provided in Supplementary Table S7.

## DISCUSSION AND CONCLUSION

Transcriptional regulators like TFs, coregulators and chromatin modifiers have a strong influence on biological processes and signaling pathways. Alterations in their activities can cause diseases like diabetes or cancer (2). Hence, understanding the role of regulators in natural and pathological processes may be the key for the detection of novel biomarkers and may even lead to the discovery of new drug targets. Therefore, the identification of transcriptional regulators that heavily influence biological processes is of utmost importance. Over the last few years, a variety of methods have been proposed that try to tackle this problem. Most of them are provided as standalone applications, but some web servers are also available: (i) *TFactS* (14) uses the hypergeometric test to detect regulators whose targets have a significant overlap with an uploaded gene list. (ii) *PASTAA* (28) computes binding affinities of TFs based on PEMs and uses the hypergeometric test to identify coregulated target genes. (iii) *Regulatory Snapshots* (22) unveils regulatory modules in expression time series data.

Here, we presented RegulatorTrail, the first web service that provides a comprehensive selection of methods for the identification of important regulators. In contrast to other approaches that have been tailored to a specific application scenario, we designed RegulatorTrail as a framework for the identification of key transcriptional regulators. It already offers eight methods for this task, and due to its modular design, it can be easily extended with further functionality. The web service can be used in four distinct application scenarios to either analyze gene lists, gene expression data or epigenetic data. Additionally, our web server is tightly connected to its sister project GeneTrail2 that can be used for downstream analysis to perform enrichment or network analysis in order to find shared mechanisms or mutually regulated signaling pathways.

In the near future, we will extend RegulatorTrail by incorporating additional methods for assessing the relevance of transcriptional regulators. Moreover, we will integrate more sophisticated methods for the assignment of regulators to their target genes. Although recent studies, see e.g. (76), confirmed that the TF binding to regulatory regions strongly influences the expression of the ‘nearest’ genes, the assignment of regulators to their target genes based only on distance information is, of course, a simplified approach that can lead to many false positive and negative RTIs. In the future, chromosome conformation capturing techniques like Hi-C may enable a cell state specific (dynamic) assignment of RTIs, see e.g. (77).

The presented case studies demonstrate the capabilities of RegulatorTrail. We were able to detect meaningful regulators that might explain the increased malignancy of metastatic melanoma compared to primary tumors as well as important regulators in macrophages. The rich functionality of our web server combined with the intuitive web interface and the well-documented RESTful API make RegulatorTrail a valuable tool for the elucidation of complex regulatory mechanisms and set it apart from other approaches.

## Supplementary Material

Supplementary Data

## References

[1] Vaquerizas J.M., Kummerfeld S.K., Teichmann S.A., Luscombe N.M. A census of human transcription factors: function, expression and evolution. Nat. Rev. Genet. 2009; 10:252–263. 19274049 10.1038/nrg2538

[2] Lee T.I., Young R.A. Transcriptional regulation and its misregulation in disease. Cell. 2013; 152:1237–1251. 23498934 10.1016/j.cell.2013.02.014 PMC3640494

[3] Latchman D.S. Transcription factors: an overview. Int. J. Biochem. Cell Biol. 1997; 29:1305–1312. 9570129 10.1016/s1357-2725(97)00085-x

[4] Maestro M.A., Cardalda C., Boj S.F., Luco R.F., Servitja J.M., Ferrer J. Distinct roles of HNF1beta, HNF1alpha, and HNF4alpha in regulating pancreas development, beta-cell function and growth. Endocr. Dev. 2007; 12:33–45. 17923767 10.1159/000109603

[5] Al-Quobaili F., Montenarh M. Pancreatic duodenal homeobox factor-1and diabetes mellitus type 2 (Review). Int. J. Mol. Med. 2008; 21:399–404. 18360684

[6] Ell B., Kang Y. Transcriptional control of cancer metastasis. Trends Cell Biol. 2013; 23:603–611. 23838335 10.1016/j.tcb.2013.06.001 PMC3815486

[7] Lerebours F., Vacher S., Andrieu C., Espie M., Marty M., Lidereau R., Bieche I. NF-kappa B genes have a major role in Inflammatory Breast Cancer. BMC Cancer. 2008; 8:41. 18248671 10.1186/1471-2407-8-41 PMC2267801

[8] Maier H.J., Schmidt-Straßburger U., Huber M.A., Wiedemann E.M., Beug H., Wirth T. NF-κB promotes epithelial–mesenchymal transition, migration and invasion of pancreatic carcinoma cells. Cancer Lett. 2010; 295:214–228. 20350779 10.1016/j.canlet.2010.03.003

[9] Chen W. NF-kappaB in lung cancer, a carcinogenesis mediator and a prevention and therapy target. Front. Biosci. 2011; 16:1172–1185. 10.2741/3782 PMC3032584 21196225

[10] Nebert D.W. Transcription factors and cancer: an overview. Toxicology. 2002; 181-182:131–141. 12505298 10.1016/s0300-483x(02)00269-x

[11] Muller P.A., Vousden K.H. Mutant p53 in cancer: new functions and therapeutic opportunities. Cancer Cell. 2014; 25:304–317. 24651012 10.1016/j.ccr.2014.01.021 PMC3970583

[12] Darnell J.E. Transcription factors as targets for cancer therapy. Nat. Rev. Cancer. 2002; 2:740–749. 12360277 10.1038/nrc906

[13] Bhagwat A.S., Vakoc C.R. Targeting transcription factors in cancer. Trends Cancer. 2015; 1:53–65. 26645049 10.1016/j.trecan.2015.07.001 PMC4669894

[14] Yeh J.E., Toniolo P.A., Frank D.A. Targeting transcription factors: promising new strategies for cancer therapy. Curr. Opin. Oncol. 2013; 25:652–658. 24048019 10.1097/01.cco.0000432528.88101.1a

[15] Essaghir A., Toffalini F., Knoops L., Kallin A., van Helden J., Demoulin J.B. Transcription factor regulation can be accurately predicted from the presence of target gene signatures in microarray gene expression data. Nucleic Acids Res. 2010; 38:e120. 20215436 10.1093/nar/gkq149 PMC2887972

[16] Yang J., Yu H., Liu B.-H., Zhao Z., Liu L., Ma L.-X., Li Y.-X., Li Y.-Y. DCGL v2.0: an R package for unveiling differential regulation from differential co-expression. PLoS One. 2013; 8:e79729. 24278165 10.1371/journal.pone.0079729 PMC3835854

[17] Reverter A., Hudson N.J., Nagaraj S.H., Perez-Enciso M., Dalrymple B.P. Regulatory impact factors: unraveling the transcriptional regulation of complex traits from expression data. Bioinformatics. 2010; 26:896–904. 20144946 10.1093/bioinformatics/btq051

[18] Huang C.-L., Lamb J., Chindelevitch L., Kostrowicki J., Guinney J., DeLisi C., Ziemek D. Correlation set analysis: detecting active regulators in disease populations using prior causal knowledge. BMC Bioinformatics. 2012; 13:46. 10.1186/1471-2105-13-46 PMC3382432 22443377

[19] Goncalves J.P., Francisco A.P., Mira N.P., Teixeira M.C., Sa-Correia I., Oliveira A.L., Madeira S.C. TFRank: network-based prioritization of regulatory associations underlying transcriptional responses. Bioinformatics. 2011; 27:3149–3157. 21965816 10.1093/bioinformatics/btr546

[20] Kawakami E., Nakaoka S., Ohta T., Kitano H. Weighted enrichment method for prediction of transcription regulators from transcriptome and global chromatin immunoprecipitation data. Nucleic Acids Res. 2016; 44:5010–5021. 27131787 10.1093/nar/gkw355 PMC4914117

[21] Poos A.M., Maicher A., Dieckmann A.K., Oswald M., Eils R., Kupiec M., Luke B., König R. Mixed Integer Linear Programming based machine learning approach identifies regulators of telomerase in yeast. Nucleic Acids Res. 2016; 44:e93. 26908654 10.1093/nar/gkw111 PMC4889924

[22] Gonçalves J.P., Aires R.S., Francisco A.P., Madeira S.C. Regulatory snapshots: integrative mining of regulatory modules from expression time series and regulatory networks. PLoS One. 2012; 7:e35977. 22563474 10.1371/journal.pone.0035977 PMC3341384

[23] Pique-Regi R., Degner J.F., Pai A.A., Gaffney D.J., Gilad Y., Pritchard J.K. Accurate inference of transcription factor binding from DNA sequence and chromatin accessibility data. Genome Res. 2011; 21:447–455. 21106904 10.1101/gr.112623.110 PMC3044858

[24] Gusmao E.G., Dieterich C., Zenke M., Costa I.G. Detection of active transcription factor binding sites with the combination of DNase hypersensitivity and histone modifications. Bioinformatics. 2014; 30:3143–3151. 25086003 10.1093/bioinformatics/btu519

[25] Sherwood R.I., Hashimoto T., O’Donnell C.W., Lewis S., Barkal A.A., van Hoff J.P., Karun V., Jaakkola T., Gifford D.K. Discovery of directional and nondirectional pioneer transcription factors by modeling DNase profile magnitude and shape. Nat. Biotechnol. 2014; 32:171–178. 24441470 10.1038/nbt.2798 PMC3951735

[26] Luo K., Hartemink A.J. Using DNase digestion data to accurately identify transcription factor binding sites. Pacific Symposium on Biocomputing. 2013; 2013:80–91. PMC3716004 23424114

[27] Kähärä J., Lähdesmäki H. BinDNase: a discriminatory approach for transcription factor binding prediction using DNase I hypersensitivity data. Bioinformatics. 2015; 31:2852–2859. 25957350 10.1093/bioinformatics/btv294

[28] Gusmao E.G., Allhoff M., Zenke M., Costa I.G. Analysis of computational footprinting methods for DNase sequencing experiments. Nat. Methods. 2016; 13:303–309. 26901649 10.1038/nmeth.3772

[29] Schmidt F., Gasparoni N., Gasparoni G., Gianmoena K., Cadenas C., Ebert P., Nordström K., Barann M., Sinha A., Fröhler S. Combining transcription factor binding affinities with open-chromatin data for accurate gene expression prediction. Nucleic Acids Res. 2017; 45:54–66. 27899623 10.1093/nar/gkw1061 PMC5224477

[30] Roider H.G., Manke T., O’Keeffe S., Vingron M., Haas S.A. PASTAA: identifying transcription factors associated with sets of co-regulated genes. Bioinformatics. 2009; 25:435–442. 19073590 10.1093/bioinformatics/btn627 PMC2642637

[31] Costa I.G., Roider H.G., do Rego T.G. Predicting gene expression in T cell differentiation from histone modifications and transcription factor binding affinities by linear mixture models. BMC Bioinformatics. 2011; 12:S29. 21342559 10.1186/1471-2105-12-S1-S29 PMC3044284

[32] McLeay R.C., Lesluyes T., Bailey T.L. Genome-wide in silico prediction of gene expression. Bioinformatics. 2012; 28:2789–2796. 22954627 10.1093/bioinformatics/bts529 PMC3476338

[33] Natarajan A., Yardimci G.G., Sheffield N.C., Crawford G.E., Ohler U. Predicting cell-type-specific gene expression from regions of open chromatin. Genome Res. 2012; 22:1711–1722. 22955983 10.1101/gr.135129.111 PMC3431488

[34] Budden D.M., Hurley D.G., Cursons J., Markham J.F., Davis M.J., Crampin E.J. Predicting expression: the complementary power of histone modification and transcription factor binding data. Epigenet. Chromatin. 2014; 7:1–12. 10.1186/1756-8935-7-36 PMC4258808 25489339

[35] Stöckel D., Kehl T., Trampert P., Schneider L., Backes C., Ludwig N., Gerasch A., Kaufmann M., Gessler M., Graf N. Multi-omics enrichment analysis using the GeneTrail2 web service. Bioinformatics. 2016; 32:1502–1508. 26787660 10.1093/bioinformatics/btv770

[36] Barrett T., Wilhite S.E., Ledoux P., Evangelista C., Kim I.F., Tomashevsky M., Marshall K.A., Phillippy K.H., Sherman P.M., Holko M. NCBI GEO: archive for functional genomics data sets–update. Nucleic Acids Res. 2012; 41:D991–D995. 23193258 10.1093/nar/gks1193 PMC3531084

[37] Riker A.I., Enkemann S.A., Fodstad O., Liu S., Ren S., Morris C., Xi Y., Howell P., Metge B., Samant R.S. The gene expression profiles of primary and metastatic melanoma yields a transition point of tumor progression and metastasis. BMC Med. Genomics. 2008; 1:13. 18442402 10.1186/1755-8794-1-13 PMC2408576

[38] Martens J.H., Stunnenberg H.G. BLUEPRINT: mapping human blood cell epigenomes. Haematologica. 2013; 98:1487–1489. 24091925 10.3324/haematol.2013.094243 PMC3789449

[39] Benjamini Y., Yekutieli D. The control of the false discovery rate in multiple testing under dependency. Ann Stat. 2001; 29:1165–1188.

[40] Essaghir A., Demoulin J. B. A minimal connected network of transcription factors regulated in human tumors and its application to the quest for universal cancer biomarkers. PLoS One. 2012; 7:e39666. 22761861 10.1371/journal.pone.0039666 PMC3382591

[41] Anders S., Huber W. Differential expression analysis for sequence count data. Genome Biology. 2010; 11:R116. 20979621 10.1186/gb-2010-11-10-r106 PMC3218662

[42] Robinson M.D., McCarthy D.J., Smyth G.K. edgeR: a Bioconductor package for differential expression analysis of digital gene expression data. Bioinformatics. 2009; 26:139–140. 19910308 10.1093/bioinformatics/btp616 PMC2796818

[43] Risso D., Ngai J., Speed T.P., Dudoit S. Normalization of RNA-seq data using factor analysis of control genes or samples. Nat. Biotechnol. 2014; 32:896–902. 25150836 10.1038/nbt.2931 PMC4404308

[44] Yao T., Wang Q., Zhang W., Bian A., Zhang J. Identification of genes associated with renal cell carcinoma using gene expression profiling analysis. Oncol. Lett. 2016; 12:73–78. 27347102 10.3892/ol.2016.4573 PMC4906613

[45] Ouyang Z., Zhou Q., Wong W.H. ChIP-Seq of transcription factors predicts absolute and differential gene expression in embryonic stem cells. Proc. Natl. Acad. Sci. U.S.A. 2009; 106:21521–21526. 19995984 10.1073/pnas.0904863106 PMC2789751

[46] Thomas-Chollier M., Hufton A., Heinig M., O’keeffe S., El Masri N., Roider H. G., Manke T., Vingron M. Transcription factor binding predictions using TRAP for the analysis of ChIP-seq data and regulatory SNPs. Nat. Protoc. 2011; 6:1860–1869. 22051799 10.1038/nprot.2011.409

[47] Consortium T.U. UniProt: the universal protein knowledgebase. Nucleic Acids Res. 2016; 45:D158–D169. 27899622 10.1093/nar/gkw1099 PMC5210571

[48] Coordinators N.R. Database Resources of the National Center for Biotechnology Information. Nucleic Acids Res. 2016; 45:D12–D17. 27899561 10.1093/nar/gkw1071 PMC5210554

[49] Lachmann A., Xu H., Krishnan J., Berger S.I., Mazloom A.R., Ma’ayan A. ChEA: transcription factor regulation inferred from integrating genome-wide ChIP-X experiments. Bioinformatics. 2010; 26:2438–2444. 20709693 10.1093/bioinformatics/btq466 PMC2944209

[50] Yang J.H., Li J.H., Jiang S., Zhou H., Qu L.H. ChIPBase: a database for decoding the transcriptional regulation of long non-coding RNA and microRNA genes from ChIP-Seq data. Nucleic Acids Res. 2012; 41:D177–D187. 23161675 10.1093/nar/gks1060 PMC3531181

[51] Sloan C.A., Chan E.T., Davidson J.M., Malladi V.S., Strattan J.S., Hitz B.C., Gabdank I., Narayanan A.K., Ho M., Lee B.T. ENCODE data at the ENCODE portal. Nucleic Acids Res. 2016; 44:D726–D732. 26527727 10.1093/nar/gkv1160 PMC4702836

[52] Mathelier A., Fornes O., Arenillas D.J., Chen C.-Y., Denay G., Lee J., Shi W., Shyr C., Tan G., Worsley-Hunt R. JASPAR 2016: a major expansion and update of the open-access database of transcription factor binding profiles. Nucleic Acids Res. 2016; 44:D110–D115. 26531826 10.1093/nar/gkv1176 PMC4702842

[53] Fazekas D., Koltai M., Türei D., Módos D., Pálfy M., Dúl Z., Zsákai L., Szalay-Bekő M., Lenti K., Farkas I.J. SignaLink 2—a signaling pathway resource with multi-layered regulatory networks. BMC Syst. Biol. 2013; 7:7. 23331499 10.1186/1752-0509-7-7 PMC3599410

[54] Matys V., Fricke E., Geffers R., Gössling E., Haubrock M., Hehl R., Hornischer K., Karas D., Kel A.E., Kel-Margoulis O.V. TRANSFAC(R): transcriptional regulation, from patterns to profiles. Nucleic Acids Res. 2003; 31:374–378. 12520026 10.1093/nar/gkg108 PMC165555

[55] Kulakovskiy I.V., Vorontsov I.E., Yevshin I.S., Soboleva A.V., Kasianov A.S., Ashoor H., Ba-alawi W., Bajic V.B., Medvedeva Y.A., Kolpakov F.A. HOCOMOCO: expansion and enhancement of the collection of transcription factor binding sites models. Nucleic Acids Res. 2016; 44:D116–D125. 26586801 10.1093/nar/gkv1249 PMC4702883

[56] Kheradpour P., Kellis M. Systematic discovery and characterization of regulatory motifs in ENCODE TF binding experiments. Nucleic Acids Res. 2013; 42:2976–2987. 24335146 10.1093/nar/gkt1249 PMC3950668

[57] Berg O. G., von Hippel P. H. Selection of DNA binding sites by regulatory proteins: Statistical-mechanical theory and application to operators and promoters. J. Mol. Biol. 1987; 193:723–743. 3612791 10.1016/0022-2836(87)90354-8

[58] Aken B.L., Achuthan P., Akanni W., Amode M.R., Bernsdorff F., Bhai J., Billis K., Carvalho-Silva D., Cummins C., Clapham P. Ensembl 2017. Nucleic Acids Res. 2017; 45:D635–D642. 27899575 10.1093/nar/gkw1104 PMC5210575

[59] GENCODE: the reference human genome annotation for The ENCODE Project. Genome Res. 2012; 22:1760–1774. 22955987 10.1101/gr.135350.111 PMC3431492

[60] Karolchik D., Hinrichs A. S., Furey T. S., Roskin K. M., Sugnet C. W., Haussler D., Kent W. J. The UCSC Table Browser data retrieval tool. Nucleic Acids Res. 2004; 32:D493–D496. 14681465 10.1093/nar/gkh103 PMC308837

[61] Hodi F.S., O’Day S.J., McDermott D.F., Weber R.W., Sosman J.A., Haanen J.B., Gonzalez R., Robert C., Schadendorf D., Hassel J.C. Improved survival with ipilimumab in patients with metastatic melanoma. N. Engl. J. Med. 2010; 363:711–723. 20525992 10.1056/NEJMoa1003466 PMC3549297

[62] Opgen-Rhein R., Strimmer K. Accurate ranking of differentially expressed genes by a distribution-free shrinkage approach. Stat. Appl. Genet. Mol. Biol. 2007; 6, doi:10.2202/1544-6115.1252. 10.2202/1544-6115.1252 17402924

[63] Liu X.S., Genet M.D., Haines J.E., Mehanna E.K., Wu S., Chen H.I., Chen Y., Qureshi A.A., Han J., Chen X. ZBTB7A suppresses melanoma metastasis by transcriptionally repressing MCAM. Mol. Cancer Res. 2015; 13:1206–1217. 25995384 10.1158/1541-7786.MCR-15-0169 PMC4543565

[64] Garraway L.A., Widlund H.R., Rubin M.A., Getz G., Berger A.J., Ramaswamy S., Beroukhim R., Milner D.A., Granter S.R., Du J. Integrative genomic analyses identify MITF as a lineage survival oncogene amplified in malignant melanoma. Nature. 2005; 436:117–122. 16001072 10.1038/nature03664

[65] Bhoumik A., Huang T.-G., Ivanov V., Gangi L., Qiao R.F., Woo S.L.C., Chen S.-H., Ronai Z. An ATF2-derived peptide sensitizes melanomas to apoptosis and inhibits their growth and metastasis. J. Clin. Invest. 2002; 110:643–650. 12208865 10.1172/JCI16081 PMC151112

[66] Bhoumik A., Gangi L., Ronai Z. Inhibition of melanoma growth and metastasis by ATF2-derived peptides. Cancer Res. 2004; 64:8222–8230. 15548688 10.1158/0008-5472.CAN-04-0714

[67] Chou J., Lin J. H., Brenot A., Kim J. W., Provot S., Werb Z. GATA3 suppresses metastasis and modulates the tumour microenvironment by regulating microRNA-29b expression. Nat. Cell Biol. 2013; 15:201–213. 23354167 10.1038/ncb2672 PMC3660859

[68] Shi D. B., Wang Y. W., Xing A. Y., Gao J. W., Zhang H., Guo X. Y., Gao P. C/EBPα-induced miR-100 expression suppresses tumor metastasis and growth by targeting ZBTB7A in gastric cancer. Cancer Lett. 2015; 369:376–385. 26404754 10.1016/j.canlet.2015.08.029

[69] Matin R.N., Chikh A., Law Pak Chong S., Mesher D., Graf Sanza' M. P., Senatore V., Scatolini M., Moretti F., Leigh I.M. p63 is an alternative p53 repressor in melanoma that confers chemoresistance and a poor prognosis. J. Exp. Med. 2013; 210:581–603. 23420876 10.1084/jem.20121439 PMC3600906

[70] Nature Immunology, Editorial A complex cell. Nat. Immunol. 2015; 17:1. 10.1038/ni.3351 26681455

[71] Al Sadoun H., Burgess M., Hentges K. E., Mace K. A. Enforced expression of Hoxa3 inhibits classical and promotes alternative activation of macrophages in vitro and in vivo. J. Immunol. 2016; 197:872–884. 27342843 10.4049/jimmunol.1501944 PMC4947829

[72] Lahouassa H., Blondot M.-L., Chauveau L., Chougui G., Morel M., Leduc M., Guillonneau F., Ramirez B.C., Schwartz O., Margottin-Goguet F. HIV-1 Vpr degrades the HLTF DNA translocase in T cells and macrophages. Proc. Natl. Acad. Sci. U.S.A. 2016; 113:5311–5316. 27114546 10.1073/pnas.1600485113 PMC4868422

[73] Zabuawala T., Taffany D. A., Sharma S. M., Merchant A., Adair B., Srinivasan R., Rosol T.J., Fernandez S., Huang K., Leone G. An ets2-driven transcriptional program in tumor-associated macrophages promotes tumor metastasis. Cancer Res. 2010; 70:1323–1333. 20145133 10.1158/0008-5472.CAN-09-1474 PMC2822898

[74] Schuetz A., Nana D., Rose C., Zocher G., Milanovic M., Koenigsmann J., Blasig R., Heinemann U., Carstanjen D. The structure of the Klf4 DNA-binding domain links to self-renewal and macrophage differentiation. Cell. Mol. Life Sci. 2011; 68:3121–3131. 21290164 10.1007/s00018-010-0618-x PMC11114807

[75] Liao X., Sharma N., Kapadia F., Zhou G., Lu Y., Hong H., Paruchuri K., Mahabeleshwar G.H., Dalmas E., Venteclef N. Krüppel-like factor 4 regulates macrophage polarization. J. Clin. Invest. 2011; 121:2736–2749. 21670502 10.1172/JCI45444 PMC3223832

[76] Ernst J., Kheradpour P., Mikkelsen T. S., Shoresh N., Ward L. D., Epstein C. B., Zhang X., Wang L., Issner R., Coyne M. Mapping and analysis of chromatin state dynamics in nine human cell types. Nature. 2011; 473:43–49. 21441907 10.1038/nature09906 PMC3088773

[77] González A. J., Setty M., Leslie C. S. Early enhancer establishment and regulatory locus complexity shape transcriptional programs in hematopoietic differentiation. Nat. Genet. 2015; 47:1249–1259. 26390058 10.1038/ng.3402 PMC4626279

