# Supplementary material for: RegulatorTrail: a web service for the identification of key transcriptional regulators
Source: Nucleic Acids Res. 2017 May 2;45(Web Server issue):W146–53. doi: 10.1093/nar/gkx350 (PMC5570139; doi:10.1093/nar/gkx350)
Supplement: Supplementary Data [file gkx350_Supp.pdf]

# RegulatorTrail: a web service for the identification of key transcriptional regulators

Tim Kehl[1,*], Lara Schneider[1], Florian Schmidt[1,2,3], Daniel Stöckel[1], Nico Gerstner[1], Christina Backes[1], Eckart Meese[1,4], Andreas Keller[1], Marcel H. Schulz[1,2,3], Hans-Peter Lenhof[1]

[1]Center for Bioinformatics, Saarland Informatics Campus, Saarland University, Saarbrücken, Germany
[2]Cluster of Excellence Multimodal Computing and Interaction, Saarland Informatics Campus, Saarland University, Saarbrücken, Germany
[3]Max Planck Institute for Informatics, Saarland Informatics Campus, Saarbrücken, Germany
[4]Human Genetics, Saarland University, Homburg, Germany

[*]To whom correspondence should be addressed. Email: tkehl@bioinf.uni-sb.de

## Supplementary Table 1 – Alternative approaches

| Name | Input | Output | Availability |
|---|---|---|---|
| TFACTS (14) | List of differentially expressed genes and collection of RTIs | Regulators with significantly over-represented target genes in the input list (p-value). | Webserver: http://www.tfacts.org/ |
| DCGL v2.0 (15) | List of differentially expressed genes and collection of RTIs | Regulators with significantly over-represented target genes in the input list (p-value). | R-package: https://cran.r-project.org/web/packages/DCGL/DCGL.pdf |
| Regulator Impact Factors RIF1 + RIF2 (16) | A gene expression matrix with samples that belong to two groups and a collection of RTIs. | Impact score for each regulator. | - |
| Correlation set analysis (17) | A gene expression matrix and a collection of RTIs. | Regulators with a significant coherence between all target genes. (p-value) | - |
| REGGAE (18) | A gene expression matrix and a collection of RTIs | A prioritized list of influential regulators. (p-value) | Webserver: https://regulatortail.bioinf.uni-sb.de |
| TFRank (19) | A list of target genes (e.g. differentially expressed genes) and a collection of weighted RTIs in form of an adjacency list (network) | A prioritized / ranked list of influential regulators | Java implementation: http://web.tecnico.ulisboa.pt/aplf/code/tfrank/ + Adapted version of the algorithm in the Regulatory Snapshots webserver (22) |
| wPGSA (20) | A score list (e.g. fold-changes) and RTIs weighted by the number of experiments that confirm each RTI | A prioritized list of influential regulators (p-value) | Python script: https://github.com/eiryo-kawakami/wPGSA |
| MIPRIP (21) | A gene expression matrix and weighted RTIs in form of an adjacency matrix (network) | A list of significant regulators per target gene (p-value) | R-package: http://www.leibniz-hki.de/de/miprip.html |
| Regulatoy Snapshots (22) | Gene expression time series and RTIs in form of regulatory networks | Important regulatory modules | Webserver: http://kdbio.inesc-id.pt/software/regulatorysnapshots/ |

Table 1A: Overview of methods based on regulator-target interactions (RTIs).

| Name | Input | Output | Availability |
|---|---|---|---|
| CENTIPEDE (23) | Matrix of read counts around candidate binding sites from DNaseI cut sites and/or histone marks | Posterior probabilities for all provided candidate binding sites to be true TF binding sites | R-package: http://centipede.uchicago.edu/ |
| PIQ (24) | Bam file with DNaseI data, and a collection of PWMs | List of purely PWM based motif matches and a list of motif matches incorporating the DNaseI information as well as the corresponding scores | R-package: https://bitbucket.org/thashim/piq-single |
| MILLIPEDE (25) | Binned DNase data, TF binding information using PWM scores, optional: TF ChIP-seq labels | Probabilities for all candidate binding sites to be true TF binding sites | R-package: https://users.cs.duke.edu/~amink/software/millipede/ |
| BinDNase (26) | Training data contains PWM scores for bound and unbound regions and DNaseI data, ideally in perBase resolution. Test data is composed of PWM scores and DnaseI data for all candidate regions | Probabilities for all candidate binding sites to be true TF binding sites | R-package: http://research.ics.aalto.fi/csb/software/bindnase/ |
| HINT-BC (27) | BED file with regions of interest, e.g. DHS sites, a Bam file with DNaseI data, and optionally Bam files with histone mark data | A Bed file with all predicted TF-footprints and the related scores | Part of the Regulatory Genomics Toolbox http://www.regulatory-genomics.org/rgt/basic-introduction/ |
| TEPIC (28) | Candidate regions for TF binding, e.g. DHS sites, a collection of PCMs, and a reference genome. Optionally, a wig file with DNaseI signal information | A file containing TF affinities calculated per peak and a file containing TF affinities calculated per gene | Bash and Python scripts as well as C++ code https://github.com/SchulzLab/TFAnalysis |
| PASTAA (29) | Ensembl IDs, Gene Symbols, or RefSeq IDs for Mouse or Human | List of TFs ranked according to the association with the input | Webserver: http://trap.molgen.mpg.de/cgi-bin/pastaa.cgi C++ Code: http://trap.molgen.mpg.de/PASTAA/ |

Table 1B: Overview of methods based on genome-wide transcription factor (TF) binding predictions.

## Supplementary Table 2 – Expected runtimes

| | Run 1 [ms] | Run 2 [ms] | Run 3 [ms] | Run 4 [ms] | Run 5 [ms] | Average runtime in *s* |
|---|---|---|---|---|---|---|
| **Hypergeometric test** | 23724 | 23793 | 23644 | 23644 | 23577 | **23.68** |
| **Fisher's exact test** | 25281 | 25409 | 25485 | 25485 | 25140 | **25.36** |
| **Binomial test** | 247275 | 247336 | 247771 | 246396 | 246948 | **247.15** |
| **REGGAE** | 172959 | 172856 | 172620 | 172620 | 172665 | **172.74** |
| **REGGAE (without bootstrapping)** | 8645 | 8415 | 8402 | 8270 | 8058 | **8.35** |
| **RIF1** | 8338 | 8108 | 8272 | 8148 | 8174 | **8.20** |
| **RIF2** | 8194 | 8115 | 8346 | 8092 | 8229 | **8.20** |

Table 2A: Average runtime of all algorithms in Scenario 1 + 2. To perform the analyses, we used GSE10072 and calculated the expression differences for tumor and normal samples. We then selected the top 250 upregulated genes for each analysis. The analyses were performed using the entire RTI collection of Database V1 2016.

| | Run 1 [ms] | Run 2 [ms] | Run 3 [ms] | Run 4 [ms] | Run 5 [ms] | Average runtime in *s* |
|---|---|---|---|---|---|---|
| **Hypergeometric test** | 28619 | 28554 | 28643 | 28709 | 28651 | **28.64** |
| **Fisher's exact test** | 32003 | 31741 | 31793 | 32069 | 31710 | **31.86** |
| **Binomial test** | 250926 | 245165 | 243075 | 243993 | 244695 | **245.57** |
| **REGGAE** | 331289 | 331978 | 345028 | 344942 | 335938 | **337.84** |
| **REGGAE (without bootstrapping)** | 8470 | 8915 | 8731 | 8744 | 8337 | **8.64** |
| **RIF1** | 8425 | 8425 | 8280 | 8111 | 8320 | **8.31** |
| **RIF2** | 8474 | 8306 | 8305 | 8341 | 8473 | **8.38** |

Table 2B: Average runtime of all algorithms in Scenario 1 + 2. To perform the analyses, we used GSE10072 and calculated the expression differences for tumor and normal samples. We then selected the top 500 upregulated genes for each analysis. The analyses were performed using the entire RTI collection of Database V1 2016.

| | **Run1 [*s*]** | **Run2 [*s*]** | **Run3 [*s*]** | **Run4 [*s*]** | **Run5 [s]** | **Average runtime in *s*** |
|---|---|---|---|---|---|---|
| **TEPIC – all PSEMs** | 469 | 471 | 471 | 476 | 470 | **471.4** |
| **TEPIC – public PSEMs** | 263 | 263 | 262 | 264 | 262 | **262.8** |
| **INVOKE lasso penalty with performance measurement** | 422 | 410 | 441 | 404 | 431 | **421.6** |
| **INVOKE lasso penalty without performance measurement** | 205 | *230* | *245* | *217* | *221* | ***224*** |
| **INVOKE ridge penalty with performance measurement** | 106 | 104 | 105 | 105 | 104 | **105** |
| **INVOKE ridge penalty without performance measurement** | *43* | *43* | *42* | *43* | *42* | ***43*** |
| **INVOKE elastic net penalty with performance measurement** | 3441 | 3606 | 3467 | 3470 | 3533 | **3503** |
| **INVOKE elastic net penalty without performance measurement** | *1683* | *1702* | *1738* | *1679* | *1772* | ***1715*** |

Table 2C: Average runtime of all algorithms in Scenario 3 + 4. To perform the analyses, we used BLUEPRINT Sample S001S7. We calculated binding affinities using TEPIC and trained predictive models of gene expression using INVOKE.

# Supplementary Table 3 – Methods for p-value adjustment

| Method | Reference |
|---|---|
| **Bonferroni** | [S1.01] |
| **Šidák** | [S1.02] |
| **Holm** | [S1.03] |
| **Holm- Šidák** | [S1.02, S1.03] |
| **Finner** | [S1.04] |
| **Hochberg** | [S1.05] |
| **Benjamini-Hochberg** | [S1.06] |
| **Benjamini-Yekutieli** | [S1.07] |


[S1.01] Bonferroni, Carlo E. Il calcolo delle assicurazioni su gruppi di teste. Tipografia del Senato, 1935.

[S1.02] Šidák, Zbyněk. "Rectangular confidence regions for the means of multivariate normal distributions." Journal of the American Statistical Association 62.318 (1967): 626-633.

[S1.03] Holm, Sture. "A simple sequentially rejective multiple test procedure." Scandinavian journal of statistics (1979): 65-70.

[S1.04] Finner, Helmut. "Some new inequalities for the range distribution, with application to the determination of optimum significance levels of multiple range tests." Journal of the American Statistical Association 85.409 (1990): 191-194.

[S1.05] Hochberg, Yosef. "A sharper Bonferroni procedure for multiple tests of significance." Biometrika 75.4 (1988): 800-802.

[S1.06] Benjamini, Yoav, and Yosef Hochberg. "Controlling the false discovery rate: a practical and powerful approach to multiple testing." Journal of the royal statistical society. Series B (Methodological) (1995): 289-300.

[S1.07] Benjamini, Yoav, and Daniel Yekutieli. "The control of the false discovery rate in multiple testing under dependency." Annals of statistics (2001): 1165-1188.

## Supplementary Table 4 – REGGAE Parameters

| Parameter | Value |
|---|---|
| **Identifier level statistic** | Shrinkage t-test |
| **Upper-quantile filter** | 0.05 |
| **Enrichment method** | Wilcoxon rank-sum test |
| **Order of test set** | decreasingly |
| **Method to compute the influence of regulators** | Pearson correlation |
| **Scoring mode for influence scores** | Absolute correlation |
| **Method to adjust p-values** | Benjamini-Yekutieli |
| **Number of bootstrap replications** | 1000 |
| **Random seed** | 8938479563690567223 |
| **RTI collection** | Entire collection V1 2016 |

## Supplementary Table 5 – REGGAE Results

| Rank | Regulator | #Targets | Score | Adjusted p-value | Mean correlation |
|---|---|---|---|---|---|
| **1** | FOSL2 | 968 | 27.07 | 4.88e-158 | -0.319 |
| **2** | CEBPA | 861 | 26.16 | 7.70e-148 | -0.328 |
| **3** | ZBTB7A | 917 | 25.58 | 1.76e-141 | -0.318 |
| **4** | SMAD1 | 780 | 25.49 | 1.35e-140 | -0.317 |
| **5** | GATA3 | 976 | 25.09 | 3.13e-136 | -0.293 |
| **6** | E2F6 | 983 | 24.14 | 3.92e-126 | 0.314 |
| **7** | MITF | 645 | 22.06 | 2.23e-105 | 0.355 |
| **8** | FOXP1 | 813 | 21.92 | 4.92e-104 | -0.294 |
| **9** | TFAP2C | 951 | 21.11 | 1.60e-96 | -0.28 |
| **10** | RXRA | 802 | 20.81 | 7.42e-94 | -0.277 |
| **11** | CBX3 | 955 | 20.31 | 1.94e-89 | 0.301 |
| **12** | BRCA1 | 741 | 19.6 | 3.03e-83 | 0.331 |
| **13** | ATF2 | 925 | 18.98 | 4.00e-78 | 0.298 |
| **14** | HEY1 | 941 | 18.92 | 1.37e-77 | 0.279 |
| **15** | TP63 | 446 | 18.63 | 2.62e-75 | -0.303 |
| **16** | BCL11A | 682 | 17.96 | 5.36e-70 | -0.271 |
| **17** | SP1 | 1018 | 17.62 | 2.49e-67 | -0.231 |
| **18** | RAD21 | 1017 | 16.91 | 5.14e-62 | 0.242 |
| **19** | PBX1 | 624 | 16.8 | 2.85e-61 | -0.268 |
| **20** | KLF5 | 658 | 16.5 | 4.24e-59 | -0.272 |
| **21** | KDM5B | 993 | 16.42 | 1.45e-58 | -0.22 |
| **22** | GRHL2 | 396 | 14.93 | 2.20e-48 | -0.282 |
| **23** | PRDM1 | 258 | 14.67 | 9.23e-47 | -0.325 |
| **24** | NANOG | 922 | 14.49 | 1.31e-45 | -0.232 |
| **25** | ETV1 | 535 | 14.18 | 1.12e-43 | 0.318 |
| **26** | NCOA1 | 419 | 13.64 | 1.97e-40 | -0.3 |
| **27** | RBL1 | 609 | 13.24 | 4.07e-38 | 0.280 |
| **28** | NFIC | 964 | 12.98 | 1.30e-36 | -0.226 |
| **29** | SMC1A | 928 | 12.9 | 3.61e-36 | 0.246 |
| **30** | CDK8 | 878 | 12.78 | 1.49e-35 | 0.244 |
| **31** | ZMIZ1 | 860 | 12.67 | 5.94e-35 | -0.214 |
| **32** | RUNX1 | 964 | 12.59 | 1.64e-34 | -0.219 |
| **33** | ZNF750 | 415 | 12.51 | 4.32e-34 | -0.258 |
| **34** | ELL2 | 440 | 12.49 | 5.36e-34 | -0.273 |
| **35** | ATF1 | 750 | 12.39 | 1.98e-33 | 0.259 |
| **36** | NIPBL | 840 | 12.16 | 3.30e-32 | -0.207 |
| **37** | KLF4 | 384 | 12.1 | 6.60e-32 | -0.271 |
| **38** | STAT3 | 988 | 11.78 | 3.03e-30 | -0.213 |
| **39** | RB1 | 707 | 11.77 | 3.17e-30 | 0.254 |
| **40** | SAP30 | 740 | 11.2 | 2.34e-27 | 0.255 |

| | | | | | |
|---|---|---|---|---|---|
| **41** | AR | 1010 | 10.84 | 1.23e-25 | -0.214 |
| **42** | PRAME | 426 | 10.81 | 1.72e-25 | 0.279 |
| **43** | ASUN | 498 | 10.76 | 2.66e-25 | 0.283 |
| **44** | YAP1 | 774 | 10.72 | 4.00e-25 | -0.206 |
| **45** | TBL1X | 435 | 10.54 | 2.70e-24 | -0.259 |
| **46** | FOXM1 | 922 | 10.5 | 4.26e-24 | 0.227 |
| **47** | CBFB | 871 | 9.807 | 5.05e-21 | 0.241 |
| **48** | NME1 | 232 | 9.66 | 2.10e-20 | 0.259 |
| **49** | RCOR1 | 967 | 9.596 | 3.83e-20 | -0.129 |
| **50** | LIN9 | 247 | 9.546 | 6.09e-20 | 0.313 |
| **51** | BCL6 | 892 | 9.502 | 9.13e-20 | -0.219 |
| **52** | LDB1 | 231 | 9.184 | 1.80e-18 | -0.258 |
| **53** | CTBP2 | 412 | 9.162 | 2.16e-18 | 0.261 |
| **54** | HOXA4 | 741 | 9.142 | 2.55e-18 | 0.251 |
| **55** | GATAD1 | 536 | 9.102 | 3.63e-18 | 0.263 |
| **56** | CHD8 | 937 | 8.904 | 2.17e-17 | -0.184 |
| **57** | EREG | 243 | 8.848 | 3.51e-17 | -0.235 |
| **58** | BCLAF1 | 806 | 8.815 | 4.66e-17 | -0.157 |
| **59** | NOTCH1 | 858 | 8.762 | 7.33e-17 | -0.193 |
| **60** | THAP1 | 392 | 8.714 | 1.10e-16 | 0.278 |
| **61** | HDAC3 | 120 | 8.471 | 9.02e-16 | -0.292 |
| **62** | WRNIP1 | 588 | 8.146 | 1.37e-14 | 0.234 |
| **63** | NCAPG | 126 | 7.988 | 4.91e-14 | 0.343 |
| **64** | RFX5 | 915 | 7.951 | 6.53e-14 | 0.201 |
| **65** | E2F1 | 942 | 7.69 | 5.10e-13 | 0.207 |
| **66** | EZH2 | 913 | 7.614 | 9.09e-13 | 0.184 |
| **67** | APOBEC3B | 164 | 7.549 | 1.48e-12 | 0.281 |
| **68** | CEBPB | 1024 | 7.324 | 7.97e-12 | -0.177 |
| **69** | TP73 | 241 | 7.114 | 3.68e-11 | -0.223 |
| **70** | TET3 | 95 | 7.026 | 6.85e-11 | -0.305 |
| **71** | RARG | 189 | 6.918 | 1.45e-10 | -0.255 |
| **72** | GREB1 | 83 | 6.884 | 1.82e-10 | 0.323 |
| **73** | RUNX3 | 969 | 6.819 | 2.83e-10 | 0.160 |
| **74** | TCF4 | 361 | 6.783 | 3.59e-10 | -0.199 |
| **75** | SUMO2 | 944 | 6.592 | 1.30e-9 | 0.161 |
| **76** | KDM6B | 780 | 6.489 | 2.56e-9 | -0.197 |
| **77** | ING2 | 842 | 6.44 | 3.49e-9 | -0.177 |
| **78** | MAF | 64 | 6.437 | 3.51e-9 | -0.307 |
| **79** | TET2 | 78 | 6.214 | 1.47e-8 | -0.277 |
| **80** | HOXB7 | 125 | 6.129 | 2.48e-8 | 0.272 |
| **81** | VDR | 632 | 5.947 | 7.60e-8 | -0.188 |
| **82** | SOX10 | 41 | 5.939 | 7.89e-8 | 0.343 |
| **83** | SCLY | 89 | 5.417 | 1.65e-6 | 0.221 |
| **84** | ZNF143 | 971 | 5.329 | 2.64e-6 | 0.189 |

| | | | | | |
|---|---|---|---|---|---|
| **85** | FOXO3 | 30 | 5.319 | 2.76e-6 | -0.317 |
| **86** | BRD3 | 755 | 5.283 | 3.32e-6 | -0.158 |
| **87** | LMNA | 428 | 5.268 | 3.57e-6 | -0.2 |
| **88** | HDAC8 | 63 | 5.134 | 7.25e-6 | 0.302 |
| **89** | GRHL1 | 36 | 5.053 | 1.10e-5 | -0.339 |
| **90** | JMJD6 | 84 | 5.045 | 1.14e-5 | 0.287 |
| **91** | CHD2 | 966 | 4.946 | 1.88e-5 | -0.132 |
| **92** | E2F7 | 223 | 4.906 | 2.28e-5 | 0.240 |
| **93** | SRF | 975 | 4.863 | 2.79e-5 | -0.175 |
| **94** | GRHL3 | 37 | 4.599 | 1.02e-4 | -0.314 |
| **95** | KDM4C | 791 | 4.583 | 1.09e-4 | -0.205 |
| **96** | ETV5 | 448 | 4.315 | 3.74e-4 | 0.224 |
| **97** | REST | 1006 | 4.277 | 4.40e-4 | -0.101 |
| **98** | BMI1 | 498 | 4.272 | 4.45e-4 | 0.204 |
| **99** | MECP2 | 509 | 4.247 | 4.93e-4 | 0.186 |
| **100** | TLE3 | 44 | 4.091 | 9.68e-4 | -0.277 |
| **101** | TFAP2A | 953 | 4.007 | 0.001 | 0.125 |
| **102** | SOX9 | 586 | 4.002 | 0.001 | -0.171 |
| **103** | FOXO1 | 385 | 3.982 | 0.001 | -0.193 |
| **104** | MAFF | 894 | 3.96 | 0.002 | 0.165 |
| **105** | ORC1 | 42 | 3.898 | 0.002 | 0.315 |
| **106** | MBD2 | 72 | 3.809 | 0.003 | -0.246 |
| **107** | NCAPG2 | 48 | 3.783 | 0.003 | 0.298 |
| **108** | TAF2 | 126 | 3.717 | 0.004 | 0.263 |
| **109** | CREBBP | 964 | 3.703 | 0.004 | -0.034 |
| **110** | RREB1 | 40 | 3.702 | 0.004 | -0.28 |
| **111** | MAFB | 87 | 3.643 | 0.005 | -0.242 |
| **112** | PRKDC | 108 | 3.608 | 0.006 | 0.249 |
| **113** | CREB1 | 988 | 3.581 | 0.007 | -0.099 |
| **114** | AUTS2 | 59 | 3.568 | 0.007 | -0.239 |
| **115** | CDK9 | 868 | 3.565 | 0.007 | -0.172 |
| **116** | GABPA | 970 | 3.542 | 0.008 | 0.116 |
| **117** | NELFE | 779 | 3.448 | 0.011 | 0.184 |
| **118** | SP2 | 343 | 3.366 | 0.015 | 0.201 |
| **119** | ZNF384 | 922 | 3.262 | 0.021 | 0.169 |
| **120** | UBTF | 756 | 3.062 | 0.041 | -0.145 |
| **121** | ELF1 | 995 | 2.978 | 0.054 | -0.13 |
| **122** | SUMO1 | 452 | 2.972 | 0.055 | 0.149 |
| **123** | NR4A1 | 148 | 2.968 | 0.055 | 0.225 |
| **124** | TERF1 | 226 | 2.919 | 0.064 | 0.231 |
| **125** | DEK | 147 | 2.855 | 0.078 | 0.230 |
| **126** | GMEB2 | 238 | 2.846 | 0.079 | -0.207 |
| **127** | ELF5 | 71 | 2.833 | 0.082 | -0.224 |
| **128** | CDK7 | 799 | 2.805 | 0.089 | -0.143 |

| | | | | | |
|---|---|---|---|---|---|
| **129** | EHF | 15 | 2.795 | 0.091 | -0.286 |
| **130** | BACH2 | 190 | 2.763 | 0.099 | -0.203 |
| **131** | OVOL2 | 21 | 2.759 | 0.099 | -0.288 |
| **132** | ASH2L | 283 | 2.758 | 0.099 | 0.221 |
| **133** | HLF | 17 | 2.72 | 0.111 | -0.21 |
| **134** | PHF8 | 959 | 2.604 | 0.155 | 0.136 |
| **135** | ZNF711 | 131 | 2.597 | 0.157 | -0.136 |
| **136** | YBX1 | 41 | 2.586 | 0.161 | 0.259 |
| **137** | ZNF83 | 320 | 2.467 | 0.224 | -0.221 |
| **138** | MYB | 867 | 2.463 | 0.225 | -0.186 |
| **139** | MBD1 | 4 | 2.447 | 0.234 | -0.257 |
| **140** | GTF2B | 817 | 2.442 | 0.235 | 0.182 |
| **141** | CBX8 | 14 | 2.403 | 0.259 | 0.313 |
| **142** | BRIP1 | 7 | 2.402 | 0.259 | 0.333 |
| **143** | YY1 | 1012 | 2.384 | 0.269 | 0.072 |
| **144** | ETS1 | 955 | 2.333 | 0.307 | 0.154 |
| **145** | NR3C1 | 980 | 2.279 | 0.352 | -0.156 |
| **146** | NCOA3 | 44 | 2.196 | 0.433 | -0.259 |
| **147** | ETV4 | 17 | 2.104 | 0.540 | 0.212 |
| **148** | E2F4 | 991 | 2.102 | 0.540 | -0.133 |
| **149** | JUNB | 658 | 2.1 | 0.540 | -0.166 |
| **150** | ZNF92 | 156 | 2.052 | 0.603 | 0.175 |
| **151** | ARNT2 | 54 | 2.037 | 0.621 | 0.264 |
| **152** | USF2 | 800 | 1.972 | 0.720 | 0.063 |
| **153** | E2F8 | 228 | 1.965 | 0.728 | 0.224 |
| **154** | CHD7 | 34 | 1.935 | 0.775 | 0.267 |
| **155** | SPDEF | 257 | 1.927 | 0.784 | -0.177 |
| **156** | SMAD3 | 730 | 1.924 | 0.786 | -0.148 |
| **157** | AREG | 37 | 1.877 | 0.868 | -0.175 |
| **158** | ING4 | 2 | 1.867 | 0.883 | -0.363 |
| **159** | DNMT1 | 11 | 1.836 | 0.941 | 0.312 |
| **160** | DDIT3 | 2 | 1.757 | 1 | 0.624 |
| **161** | HECTD1 | 2 | 1.753 | 1 | -0.379 |
| **162** | PBX2 | 7 | 1.75 | 1 | 0.340 |
| **163** | NFATC1 | 832 | 1.749 | 1 | -0.184 |
| **164** | WDHD1 | 1 | 1.721 | 1 | 0.763 |
| **165** | DOT1L | 1 | 1.708 | 1 | 0.593 |
| **166** | RPA3 | 1 | 1.706 | 1 | 0.815 |
| **167** | ZBTB33 | 718 | 1.703 | 1 | 0.118 |
| **168** | DNAJC2 | 38 | 1.664 | 1 | 0.164 |
| **169** | TTF2 | 109 | 1.656 | 1 | 0.229 |
| **170** | MAX | 1023 | 1.648 | 1 | 0.135 |
| **171** | SFPQ | 878 | 1.628 | 1 | -0.109 |
| **172** | PHF6 | 5 | 1.607 | 1 | 0.354 |

| | | | | | |
|---|---|---|---|---|---|
| **173** | TCF7L2 | 987 | 1.564 | 1 | -0.116 |
| **174** | NFKB2 | 1 | 1.521 | 1 | 0.408 |
| **175** | ZMYND11 | 5 | 1.507 | 1 | -0.264 |
| **176** | TAF7 | 805 | 1.476 | 1 | 0.172 |
| **177** | TRRAP | 190 | 1.453 | 1 | 0.218 |
| **178** | POU3F2 | 110 | 1.438 | 1 | 0.192 |
| **179** | FOSB | 1 | 1.429 | 1 | 0.333 |
| **180** | FANCD2 | 1 | 1.421 | 1 | 0.423 |
| **181** | BDP1 | 392 | 1.42 | 1 | 0.085 |
| **182** | NR2F6 | 1 | 1.364 | 1 | -0.324 |
| **183** | ZC3H8 | 57 | 1.357 | 1 | 0.232 |
| **184** | TRIM24 | 1 | 1.335 | 1 | 0.325 |
| **185** | DEAF1 | 1 | 1.334 | 1 | -0.339 |
| **186** | NFE2 | 850 | 1.33 | 1 | -0.183 |
| **187** | PURA | 1 | 1.303 | 1 | 0.288 |
| **188** | KDM6A | 48 | 1.298 | 1 | -0.198 |
| **189** | MBD3 | 4 | 1.291 | 1 | -0.255 |
| **190** | PLSCR1 | 1 | 1.281 | 1 | 0.344 |
| **191** | NOLC1 | 1 | 1.277 | 1 | 0.415 |
| **192** | ELOF1 | 10 | 1.25 | 1 | 0.145 |
| **193** | KAT5 | 180 | 1.229 | 1 | -0.141 |
| **194** | PARP1 | 2 | 1.227 | 1 | 0.315 |
| **195** | SOX4 | 171 | 1.224 | 1 | -0.109 |
| **196** | BATF | 804 | 1.208 | 1 | 0.112 |
| **197** | HES1 | 1 | 1.2 | 1 | -0.232 |
| **198** | ELF3 | 1 | 1.152 | 1 | -0.164 |
| **199** | NR1H3 | 58 | 1.119 | 1 | 0.174 |
| **200** | DVL1 | 1 | 1.082 | 1 | 0.281 |
| **201** | ZNF486 | 19 | 1.077 | 1 | -0.198 |
| **202** | DYRK1A | 21 | 1.074 | 1 | 0.166 |
| **203** | HTT | 10 | 1.064 | 1 | -0.195 |
| **204** | HNRNPK | 1 | 1.048 | 1 | -0.098 |
| **205** | MYCN | 42 | 1.038 | 1 | -0.114 |
| **206** | HMGA2 | 1 | 1.036 | 1 | 0.363 |
| **207** | DLX3 | 1 | 1.027 | 1 | -0.242 |
| **208** | PLAU | 1 | 1.008 | 1 | 0.189 |
| **209** | RAD51 | 1 | 0.983 | 1 | 0.425 |
| **210** | CDK2 | 4 | 0.956 | 1 | 0.352 |
| **211** | ERG | 1004 | 0.954 | 1 | -0.146 |
| **212** | MIA3 | 3 | 0.908 | 1 | 0.262 |
| **213** | IRF2 | 101 | 0.894 | 1 | 0.203 |
| **214** | WHSC1 | 2 | 0.850 | 1 | 0.300 |
| **215** | ATF7 | 1 | 0.845 | 1 | -0.244 |
| **216** | RELA | 985 | 0.842 | 1 | -0.086 |

| | | | | | |
|---|---|---|---|---|---|
| **217** | NKX2-2 | 1 | 0.837 | 1 | 0.220 |
| **218** | RPA1 | 1 | 0.827 | 1 | 0.334 |
| **219** | CBX4 | 15 | 0.821 | 1 | 0.245 |
| **220** | NR2F1 | 792 | 0.808 | 1 | 0.172 |
| **221** | SP3 | 20 | 0.808 | 1 | -0.186 |
| **222** | XRCC5 | 1 | 0.803 | 1 | -0.256 |
| **223** | UBP1 | 1 | 0.784 | 1 | -0.327 |
| **224** | KLF8 | 1 | 0.776 | 1 | -0.219 |
| **225** | ATF7IP | 6 | 0.772 | 1 | -0.166 |
| **226** | GABPB1 | 1 | 0.738 | 1 | 0.312 |
| **227** | FLII | 29 | 0.706 | 1 | -0.037 |
| **228** | STAT5A | 800 | 0.690 | 1 | 0.120 |
| **229** | PIAS1 | 400 | 0.686 | 1 | -0.151 |
| **230** | LHX2 | 49 | 0.677 | 1 | -0.142 |
| **231** | CBX2 | 7 | 0.636 | 1 | 0.217 |
| **232** | HDAC2 | 956 | 0.630 | 1 | -0.078 |
| **233** | BCL11B | 1 | 0.619 | 1 | -0.228 |
| **234** | TEAD4 | 990 | 0.611 | 1 | 0.122 |
| **235** | NFATC4 | 1 | 0.610 | 1 | 0.234 |
| **236** | TEAD1 | 436 | 0.592 | 1 | 0.162 |
| **237** | THRB | 1 | 0.567 | 1 | -0.202 |
| **238** | ETS2 | 3 | 0.549 | 1 | -0.248 |
| **239** | ESRRG | 1 | 0.523 | 1 | 0.211 |
| **240** | BRD7 | 27 | 0.493 | 1 | -0.117 |
| **241** | ARRB1 | 12 | 0.492 | 1 | -0.25 |
| **242** | ZZZ3 | 257 | 0.473 | 1 | 0.157 |
| **243** | ZNF395 | 3 | 0.473 | 1 | -0.272 |
| **244** | SRCAP | 8 | 0.468 | 1 | -0.223 |
| **245** | HAND2 | 4 | 0.453 | 1 | 0.169 |
| **246** | GLI2 | 596 | 0.433 | 1 | -0.15 |
| **247** | FOXO4 | 1 | 0.431 | 1 | -0.171 |
| **248** | ARNT | 325 | 0.426 | 1 | -0.17 |
| **249** | REL | 3 | 0.413 | 1 | -0.236 |
| **250** | GDNF | 3 | 0.410 | 1 | -0.085 |
| **251** | NFE2L2 | 88 | 0.401 | 1 | -0.126 |
| **252** | ZNF350 | 1 | 0.381 | 1 | -0.166 |
| **253** | BRF2 | 232 | 0.377 | 1 | 0.158 |
| **254** | EGFR | 4 | 0.370 | 1 | -0.169 |
| **255** | KCNIP3 | 1 | 0.369 | 1 | 0.156 |
| **256** | HIF1A | 941 | 0.360 | 1 | 0.115 |
| **257** | CBX7 | 4 | 0.346 | 1 | -0.174 |
| **258** | LTF | 1 | 0.319 | 1 | -0.232 |
| **259** | DROSHA | 11 | 0.299 | 1 | 0.161 |
| **260** | ELF2 | 11 | 0.294 | 1 | -0.228 |

| | | | | | |
|---|---|---|---|---|---|
| **261** | NFKB1 | 222 | 0.280 | 1 | -0.142 |
| **262** | OLIG2 | 1 | 0.277 | 1 | 0.155 |
| **263** | NR3C2 | 2 | 0.271 | 1 | -0.273 |
| **264** | AGO1 | 232 | 0.256 | 1 | -0.187 |
| **265** | MZF1 | 2 | 0.251 | 1 | -0.202 |
| **266** | TCFL5 | 1 | 0.232 | 1 | 0.201 |
| **267** | EBF1 | 946 | 0.217 | 1 | -0.163 |
| **268** | NFYB | 775 | 0.202 | 1 | -0.052 |
| **269** | DAND5 | 3 | 0.161 | 1 | -0.064 |
| **270** | ISL2 | 2 | 0.160 | 1 | 0.089 |
| **271** | IRF5 | 40 | 0.157 | 1 | -0.058 |
| **272** | HIC1 | 1 | 0.140 | 1 | 0.012 |
| **273** | HOXC10 | 1 | 0.138 | 1 | 0.184 |
| **274** | CREM | 1 | 0.122 | 1 | 0.173 |
| **275** | NR5A2 | 28 | 0.106 | 1 | 0.159 |
| **276** | NFIB | 1 | 0.098 | 1 | -0.237 |
| **277** | FOXE1 | 3 | 0.083 | 1 | -0.103 |
| **278** | TP53BP1 | 17 | 0.081 | 1 | 0.076 |
| **279** | TDRD3 | 6 | 0.062 | 1 | 0.252 |
| **280** | PPARD | 200 | 0.030 | 1 | -0.004 |
| **281** | NFATC2 | 3 | 0.019 | 1 | 0.106 |
| **282** | TFE3 | 1 | -0.004 | 1 | 0.129 |
| **283** | ZIC1 | 1 | -0.012 | 1 | -0.147 |
| **284** | PALB2 | 9 | -0.014 | 1 | 0.226 |
| **285** | SETD2 | 2 | -0.02 | 1 | -0.15 |
| **286** | MAPK1 | 4 | -0.028 | 1 | 0.104 |
| **287** | HOXC13 | 1 | -0.034 | 1 | 0.149 |
| **288** | RFX2 | 20 | -0.036 | 1 | -0.194 |
| **289** | WDR5 | 914 | -0.06 | 1 | -0.113 |
| **290** | ETV7 | 1 | -0.061 | 1 | -0.117 |
| **291** | DR1 | 30 | -0.072 | 1 | 0.194 |
| **292** | SNAPC1 | 7 | -0.078 | 1 | 0.186 |
| **293** | HOXC11 | 1 | -0.092 | 1 | 0.125 |
| **294** | PSIP1 | 14 | -0.106 | 1 | 0.207 |
| **295** | FOXD1 | 1 | -0.132 | 1 | 0.129 |
| **296** | AGO2 | 29 | -0.132 | 1 | 0.024 |
| **297** | SNAPC2 | 1 | -0.174 | 1 | -0.06 |
| **298** | KDM5C | 53 | -0.185 | 1 | -0.148 |
| **299** | C17ORF96 | 9 | -0.2 | 1 | -0.055 |
| **300** | RYBP | 2 | -0.23 | 1 | 0.096 |
| **301** | ZIC2 | 1 | -0.279 | 1 | -0.107 |
| **302** | CHD1 | 839 | -0.298 | 1 | 0.148 |
| **303** | SFMBT1 | 12 | -0.318 | 1 | 0.141 |
| **304** | EGR2 | 24 | -0.319 | 1 | -0.063 |

| | | | | | |
|---|---|---|---|---|---|
| **305** | TFCP2 | 1 | -0.32 | 1 | 0.205 |
| **306** | TFDP1 | 2 | -0.322 | 1 | 0.192 |
| **307** | TERF2 | 2 | -0.328 | 1 | 0.100 |
| **308** | POLR2A | 1002 | -0.33 | 1 | -0.143 |
| **309** | PPARA | 5 | -0.333 | 1 | -0.059 |
| **310** | APEX1 | 1 | -0.334 | 1 | 0.176 |
| **311** | DNTTIP1 | 1 | -0.336 | 1 | 0.133 |
| **312** | DSCAM | 2 | -0.347 | 1 | -0.139 |
| **313** | EPAS1 | 50 | -0.349 | 1 | -0.104 |
| **314** | BRD4 | 959 | -0.352 | 1 | -0.124 |
| **315** | RUNX2 | 194 | -0.354 | 1 | 0.147 |
| **316** | POLR3G | 4 | -0.368 | 1 | 0.168 |
| **317** | ARNTL | 897 | -0.369 | 1 | -0.159 |
| **318** | POU2F1 | 125 | -0.378 | 1 | -0.066 |
| **319** | SPANXC | 1 | -0.384 | 1 | -0.106 |
| **320** | CIITA | 45 | -0.42 | 1 | -0.056 |
| **321** | TFAP2D | 2 | -0.424 | 1 | -0.104 |
| **322** | TOP2B | 96 | -0.431 | 1 | 0.027 |
| **323** | HOXA5 | 7 | -0.437 | 1 | 0.171 |
| **324** | INO80 | 20 | -0.441 | 1 | -0.073 |
| **325** | CKAP4 | 545 | -0.459 | 1 | -0.124 |
| **326** | PPARGC1A | 134 | -0.462 | 1 | 0.170 |
| **327** | CBFA2T3 | 3 | -0.476 | 1 | -0.093 |
| **328** | KDM2B | 1 | -0.487 | 1 | -0.15 |
| **329** | BLVRA | 1 | -0.516 | 1 | 0.143 |
| **330** | XRCC4 | 9 | -0.519 | 1 | 0.081 |
| **331** | HLTF | 1 | -0.531 | 1 | 0.084 |
| **332** | MRE11A | 57 | -0.552 | 1 | 0.124 |
| **333** | HOXA2 | 1 | -0.556 | 1 | -0.064 |
| **334** | ZBTB24 | 2 | -0.57 | 1 | 0.229 |
| **335** | SLC6A4 | 2 | -0.636 | 1 | -0.122 |
| **336** | ARHGEF7 | 9 | -0.643 | 1 | 0.072 |
| **337** | CDX1 | 1 | -0.661 | 1 | -0.175 |
| **338** | DEC1 | 1 | -0.688 | 1 | -0.108 |
| **339** | IRF9 | 2 | -0.698 | 1 | -0.115 |
| **340** | HNF1B | 11 | -0.699 | 1 | 0.049 |
| **341** | AFF4 | 704 | -0.707 | 1 | -0.003 |
| **342** | SIRT3 | 1 | -0.719 | 1 | 0.073 |
| **343** | EWSR1 | 46 | -0.72 | 1 | 0.130 |
| **344** | SORBS2 | 1 | -0.727 | 1 | -0.061 |
| **345** | TCF7L1 | 1 | -0.751 | 1 | -0.074 |
| **346** | FABP4 | 1 | -0.76 | 1 | -0.121 |
| **347** | HNRNPL | 1 | -0.805 | 1 | -0.144 |
| **348** | ARNTL2 | 1 | -0.807 | 1 | -0.028 |

| | | | | | |
|---|---|---|---|---|---|
| **349** | CARM1 | 5 | -0.841 | 1 | 0.128 |
| **350** | SNAPC5 | 3 | -0.845 | 1 | 0.157 |
| **351** | PLAG1 | 29 | -0.851 | 1 | -0.119 |
| **352** | HOXA13 | 2 | -0.851 | 1 | -0.03 |
| **353** | CTNNB1 | 261 | -0.875 | 1 | 0.167 |
| **354** | FOXK1 | 4 | -0.89 | 1 | 0.109 |
| **355** | GLI1 | 7 | -0.9 | 1 | -0.08 |
| **356** | TFF2 | 1 | -0.901 | 1 | 0.100 |
| **357** | RXRB | 1 | -0.904 | 1 | -0.016 |
| **358** | E2F2 | 7 | -0.911 | 1 | 0.059 |
| **359** | HOXA10 | 1 | -0.93 | 1 | 0.084 |
| **360** | ZNF148 | 1 | -0.933 | 1 | -0.087 |
| **361** | MTA1 | 1 | -0.947 | 1 | -0.059 |
| **362** | SALL2 | 1 | -0.952 | 1 | 0.109 |
| **363** | PES1 | 1 | -0.962 | 1 | -0.006 |
| **364** | AFF1 | 227 | -0.963 | 1 | -0.165 |
| **365** | ELL | 11 | -0.965 | 1 | -0.183 |
| **366** | ZC3H11A | 28 | -0.965 | 1 | 0.007 |
| **367** | XRN2 | 139 | -0.97 | 1 | 0.156 |
| **368** | LIN54 | 139 | -0.999 | 1 | 0.047 |
| **369** | RXRG | 1 | -1.004 | 1 | 0.069 |
| **370** | RPA2 | 1 | -1.01 | 1 | 0.099 |
| **371** | CEBPZ | 16 | -1.012 | 1 | 0.130 |
| **372** | PCGF2 | 79 | -1.043 | 1 | 0.014 |
| **373** | PIAS4 | 11 | -1.106 | 1 | -0.052 |
| **374** | HSF1 | 887 | -1.109 | 1 | 0.123 |
| **375** | GFI1 | 1 | -1.114 | 1 | -0.013 |
| **376** | PRDM2 | 1 | -1.132 | 1 | 0.020 |
| **377** | MTF1 | 1 | -1.132 | 1 | 0.043 |
| **378** | CBX6 | 4 | -1.154 | 1 | 0.089 |
| **379** | ZFHX4 | 10 | -1.176 | 1 | 0.129 |
| **380** | GTF2I | 567 | -1.179 | 1 | 0.153 |
| **381** | IRF8 | 14 | -1.185 | 1 | 0.076 |
| **382** | E2F5 | 2 | -1.186 | 1 | 0.105 |
| **383** | ZNF652 | 5 | -1.199 | 1 | 0.034 |
| **384** | FUBP1 | 1 | -1.2 | 1 | 0.064 |
| **385** | HMGA1 | 3 | -1.202 | 1 | 0.078 |
| **386** | FOXF2 | 51 | -1.211 | 1 | 0.144 |
| **387** | ELF4 | 1 | -1.216 | 1 | -0.025 |
| **388** | CBX5 | 5 | -1.226 | 1 | -0.091 |
| **389** | SCML2 | 180 | -1.228 | 1 | 0.149 |
| **390** | MEF2C | 447 | -1.231 | 1 | 0.166 |
| **391** | BCL10 | 1 | -1.244 | 1 | 0.007 |
| **392** | DPPA3 | 2 | -1.246 | 1 | -0.038 |

| | | | | | |
|---|---|---|---|---|---|
| **393** | ZNF281 | 3 | -1.247 | 1 | 0.110 |
| **394** | NR1H4 | 28 | -1.264 | 1 | 0.036 |
| **395** | CTBP1 | 263 | -1.271 | 1 | -0.159 |
| **396** | ICE2 | 10 | -1.279 | 1 | 0.068 |
| **397** | HMBOX1 | 24 | -1.302 | 1 | -0.105 |
| **398** | TFAM | 1 | -1.304 | 1 | 0.143 |
| **399** | RLF | 2 | -1.307 | 1 | 0.031 |
| **400** | T | 147 | -1.311 | 1 | 0.147 |
| **401** | SOX11 | 50 | -1.333 | 1 | 0.038 |
| **402** | ZNF12 | 23 | -1.351 | 1 | 0.218 |
| **403** | NFYC | 364 | -1.358 | 1 | -0.122 |
| **404** | INSM1 | 51 | -1.375 | 1 | 0.083 |
| **405** | GLI3 | 6 | -1.386 | 1 | -9.85e-4 |
| **406** | ASF1A | 170 | -1.395 | 1 | -0.104 |
| **407** | L3MBTL2 | 12 | -1.396 | 1 | 0.073 |
| **408** | NR5A1 | 8 | -1.4 | 1 | 0.095 |
| **409** | ZNF280D | 255 | -1.402 | 1 | 0.144 |
| **410** | HSF4 | 1 | -1.456 | 1 | -0.023 |
| **411** | ZNF84 | 21 | -1.484 | 1 | -0.021 |
| **412** | KCNH8 | 23 | -1.499 | 1 | -0.04 |
| **413** | KDM3A | 165 | -1.507 | 1 | 0.099 |
| **414** | GPS2 | 19 | -1.522 | 1 | -0.076 |
| **415** | ATRX | 3 | -1.532 | 1 | 0.068 |
| **416** | ELK1 | 646 | -1.537 | 1 | 0.100 |
| **417** | BCOR | 869 | -1.547 | 1 | 0.142 |
| **418** | ZFP42 | 95 | -1.648 | 1 | -0.172 |
| **419** | SALL1 | 5 | -1.671 | 1 | 0.041 |
| **420** | SLC22A1 | 1 | -1.692 | 1 | -0.052 |
| **421** | KMT2A | 651 | -1.708 | 1 | -0.144 |
| **422** | CLOCK | 28 | -1.727 | 1 | -0.075 |
| **423** | NRF1 | 919 | -1.742 | 1 | -0.092 |
| **424** | ZKSCAN1 | 315 | -1.747 | 1 | 0.154 |
| **425** | ERCC3 | 184 | -1.753 | 1 | 0.146 |
| **426** | NR2C2 | 501 | -1.764 | 1 | -0.078 |
| **427** | LMTK3 | 31 | -1.782 | 1 | -0.13 |
| **428** | STAT6 | 36 | -1.79 | 1 | -0.07 |
| **429** | HOXC8 | 4 | -1.798 | 1 | 0.072 |
| **430** | GBX2 | 11 | -1.803 | 1 | -0.055 |
| **431** | CDX2 | 970 | -1.81 | 1 | 0.064 |
| **432** | SVIL | 2 | -1.847 | 1 | -0.056 |
| **433** | DACH1 | 138 | -1.873 | 1 | -0.149 |
| **434** | SSRP1 | 73 | -1.928 | 1 | 0.057 |
| **435** | ZBTB10 | 106 | -1.93 | 1 | 0.174 |
| **436** | HSF2 | 4 | -1.933 | 1 | 0.002 |

| | | | | | |
|---|---|---|---|---|---|
| **437** | DCP1A | 487 | -2.001 | 1 | 0.146 |
| **438** | ZFX | 229 | -2.044 | 1 | 0.139 |
| **439** | EZH1 | 57 | -2.09 | 1 | -0.125 |
| **440** | LEF1 | 74 | -2.102 | 1 | 0.137 |
| **441** | RBL2 | 328 | -2.126 | 1 | -0.101 |
| **442** | HINFP | 3 | -2.17 | 1 | 0.080 |
| **443** | SETDB1 | 709 | -2.218 | 1 | -0.026 |
| **444** | SMAD2 | 355 | -2.235 | 1 | 0.024 |
| **445** | KAT2B | 22 | -2.249 | 1 | -0.024 |
| **446** | PRKCQ | 29 | -2.255 | 1 | -0.129 |
| **447** | NHLH1 | 39 | -2.308 | 1 | 0.060 |
| **448** | CCNT2 | 824 | -2.342 | 1 | 0.015 |
| **449** | MYC | 1026 | -2.353 | 1 | 0.134 |
| **450** | STAT4 | 93 | -2.371 | 1 | 0.031 |
| **451** | HMGN3 | 793 | -2.424 | 1 | -0.083 |
| **452** | TFAP4 | 442 | -2.467 | 1 | 0.135 |
| **453** | NR2F2 | 951 | -2.476 | 1 | 0.092 |
| **454** | TFEB | 71 | -2.484 | 1 | -0.088 |
| **455** | ONECUT1 | 30 | -2.494 | 1 | 0.077 |
| **456** | HDAC6 | 128 | -2.503 | 1 | -0.049 |
| **457** | FOXH1 | 539 | -2.505 | 1 | 0.102 |
| **458** | WWTR1 | 145 | -2.524 | 1 | 0.135 |
| **459** | KAT8 | 111 | -2.566 | 1 | -0.13 |
| **460** | MEIS1 | 58 | -2.685 | 1 | -0.092 |
| **461** | HEYL | 19 | -2.692 | 1 | 0.007 |
| **462** | LMO3 | 164 | -2.744 | 1 | -0.118 |
| **463** | MYBL2 | 819 | -2.812 | 1 | 0.148 |
| **464** | ZNF274 | 101 | -2.854 | 1 | -0.074 |
| **465** | TBP | 993 | -2.869 | 1 | -0.127 |
| **466** | GFI1B | 30 | -2.932 | 1 | -0.07 |
| **467** | TOP1 | 306 | -2.954 | 1 | 0.048 |
| **468** | ALKBH3 | 26 | -2.988 | 1 | -0.001 |
| **469** | SMC3 | 959 | -3.007 | 1 | 0.009 |
| **470** | NFYA | 821 | -3.016 | 1 | 0.142 |
| **471** | NOS2 | 12 | -3.02 | 1 | -0.005 |
| **472** | FOXI1 | 46 | -3.063 | 1 | -0.094 |
| **473** | SMARCB1 | 933 | -3.074 | 1 | 0.101 |
| **474** | HNF1A | 23 | -3.109 | 1 | -0.067 |
| **475** | TCF3 | 906 | -3.141 | 1 | 0.068 |
| **476** | NCOR2 | 337 | -3.175 | 1 | -0.122 |
| **477** | ESRRA | 225 | -3.197 | 1 | -0.051 |
| **478** | ELK4 | 484 | -3.245 | 1 | -0.114 |
| **479** | BACH1 | 653 | -3.257 | 1 | 0.053 |
| **480** | TP53 | 851 | -3.282 | 1 | -0.086 |

| | | | | | |
|---|---|---|---|---|---|
| **481** | SOX17 | 40 | -3.309 | 1 | 0.012 |
| **482** | ASCL1 | 236 | -3.326 | 1 | 0.090 |
| **483** | RUNX1T1 | 672 | -3.472 | 1 | -0.137 |
| **484** | GLYR1 | 17 | -3.484 | 1 | 0.108 |
| **485** | HIRA | 130 | -3.594 | 1 | 0.009 |
| **486** | TAL1 | 968 | -3.599 | 1 | -0.074 |
| **487** | TCF12 | 997 | -3.632 | 1 | 0.059 |
| **488** | FOXA3 | 81 | -3.719 | 1 | 0.090 |
| **489** | RNF2 | 255 | -3.77 | 1 | 0.118 |
| **490** | SIN3A | 996 | -3.796 | 1 | -0.093 |
| **491** | INTS3 | 686 | -3.811 | 1 | 0.063 |
| **492** | EED | 113 | -3.818 | 1 | 0.059 |
| **493** | MXD3 | 27 | -3.855 | 1 | -0.038 |
| **494** | HDAC1 | 870 | -3.941 | 1 | 0.118 |
| **495** | CYP27B1 | 17 | -3.962 | 1 | -0.045 |
| **496** | CTCF | 1022 | -3.974 | 1 | -0.037 |
| **497** | LMNB1 | 744 | -3.974 | 1 | 0.124 |
| **498** | HOXC9 | 170 | -4.038 | 1 | 0.108 |
| **499** | SUPT5H | 867 | -4.049 | 1 | -0.045 |
| **500** | SALL4 | 104 | -4.102 | 1 | 0.105 |
| **501** | LYL1 | 175 | -4.314 | 1 | 0.043 |
| **502** | GTF3C2 | 228 | -4.387 | 1 | 0.081 |
| **503** | ICE1 | 636 | -4.417 | 1 | 0.124 |
| **504** | STAT2 | 574 | -4.504 | 1 | 0.047 |
| **505** | PRDM14 | 89 | -4.519 | 1 | 0.042 |
| **506** | C17ORF49 | 188 | -4.521 | 1 | -0.093 |
| **507** | BRD1 | 703 | -4.569 | 1 | -0.117 |
| **508** | TBX21 | 166 | -4.693 | 1 | 0.031 |
| **509** | AHR | 210 | -4.724 | 1 | 0.020 |
| **510** | ARID3A | 745 | -4.739 | 1 | -0.003 |
| **511** | SIRT6 | 301 | -4.762 | 1 | -0.08 |
| **512** | IRF4 | 931 | -4.831 | 1 | 0.072 |
| **513** | CSNK2A1 | 148 | -4.863 | 1 | 0.101 |
| **514** | IRF3 | 167 | -4.898 | 1 | -0.005 |
| **515** | KDM5A | 188 | -4.983 | 1 | -0.085 |
| **516** | CDK12 | 99 | -5.013 | 1 | 0.059 |
| **517** | BRF1 | 339 | -5.03 | 1 | -0.068 |
| **518** | CTCFL | 919 | -5.032 | 1 | 0.003 |
| **519** | GTF2F1 | 799 | -5.065 | 1 | 0.013 |
| **520** | ERCC2 | 112 | -5.089 | 1 | -0.11 |
| **521** | HMGN1 | 112 | -5.199 | 1 | 0.039 |
| **522** | E2F3 | 712 | -5.226 | 1 | 0.149 |
| **523** | ZNF263 | 941 | -5.264 | 1 | -0.07 |
| **524** | TRIM28 | 995 | -5.321 | 1 | 0.016 |

| | | | | | |
|---|---|---|---|---|---|
| **525** | FOXA2 | 989 | -5.357 | 1 | 0.068 |
| **526** | JUN | 1010 | -5.373 | 1 | -0.082 |
| **527** | ELK3 | 362 | -5.417 | 1 | 0.077 |
| **528** | MTA3 | 786 | -5.473 | 1 | -0.15 |
| **529** | KAT2A | 247 | -5.496 | 1 | 0.063 |
| **530** | PADI4 | 98 | -5.542 | 1 | -0.114 |
| **531** | PAF1 | 131 | -5.571 | 1 | -0.01 |
| **532** | PAX6 | 218 | -5.581 | 1 | -0.076 |
| **533** | GATA2 | 969 | -5.599 | 1 | -0.112 |
| **534** | CHD4 | 151 | -5.656 | 1 | -0.03 |
| **535** | ZNF76 | 173 | -5.752 | 1 | 0.106 |
| **536** | ESR2 | 289 | -5.768 | 1 | -0.06 |
| **537** | JUND | 1018 | -5.773 | 1 | -0.075 |
| **538** | WT1 | 199 | -5.852 | 1 | 0.072 |
| **539** | TBL1XR1 | 791 | -5.859 | 1 | -7.94e-4 |
| **540** | FOSL1 | 885 | -6.022 | 1 | 0.125 |
| **541** | PDX1 | 277 | -6.065 | 1 | 3.50e-4 |
| **542** | SP4 | 631 | -6.084 | 1 | 0.082 |
| **543** | FUS | 211 | -6.093 | 1 | -0.015 |
| **544** | EOMES | 609 | -6.226 | 1 | 0.110 |
| **545** | ZNF217 | 354 | -6.261 | 1 | 0.030 |
| **546** | THAP11 | 153 | -6.345 | 1 | -0.113 |
| **547** | SUPT20H | 191 | -6.352 | 1 | 0.034 |
| **548** | BCL3 | 855 | -6.361 | 1 | -0.071 |
| **549** | POLR3A | 507 | -6.385 | 1 | -0.029 |
| **550** | IRF1 | 951 | -6.4 | 1 | -0.074 |
| **551** | SREBF1 | 557 | -6.406 | 1 | -0.107 |
| **552** | SREBF2 | 236 | -6.481 | 1 | -0.033 |
| **553** | SMARCA4 | 909 | -6.492 | 1 | 0.013 |
| **554** | MED12 | 328 | -6.56 | 1 | 0.034 |
| **555** | SMARCC1 | 867 | -6.701 | 1 | 0.006 |
| **556** | CUX1 | 851 | -6.726 | 1 | -0.007 |
| **557** | STAT5B | 350 | -6.75 | 1 | 0.025 |
| **558** | NKX3-1 | 375 | -6.775 | 1 | 0.047 |
| **559** | HNF4G | 645 | -6.791 | 1 | 0.129 |
| **560** | SNAI2 | 903 | -6.905 | 1 | -0.057 |
| **561** | DAXX | 564 | -6.959 | 1 | -0.042 |
| **562** | SMAD4 | 455 | -6.985 | 1 | -0.011 |
| **563** | EHMT2 | 301 | -7.056 | 1 | -0.005 |
| **564** | STAG1 | 886 | -7.069 | 1 | -0.054 |
| **565** | EGR1 | 984 | -7.205 | 1 | -0.104 |
| **566** | LMO2 | 320 | -7.297 | 1 | 0.009 |
| **567** | MED1 | 952 | -7.385 | 1 | -0.017 |
| **568** | ZEB1 | 776 | -7.407 | 1 | -0.006 |

| | | | | | |
|---|---|---|---|---|---|
| **569** | CPSF3L | 507 | -7.414 | 1 | -0.022 |
| **570** | MYH11 | 818 | -7.418 | 1 | -0.138 |
| **571** | RBBP5 | 967 | -7.422 | 1 | 0.052 |
| **572** | DDX5 | 914 | -7.429 | 1 | 0.026 |
| **573** | TAF3 | 821 | -7.482 | 1 | -0.084 |
| **574** | STAT1 | 992 | -7.487 | 1 | 0.067 |
| **575** | PROX1 | 192 | -7.675 | 1 | -0.056 |
| **576** | SUZ12 | 420 | -7.69 | 1 | 0.079 |
| **577** | CDK6 | 351 | -7.73 | 1 | 0.008 |
| **578** | RBPJ | 706 | -7.755 | 1 | -0.034 |
| **579** | FOXP3 | 163 | -7.764 | 1 | -0.016 |
| **580** | ZBTB17 | 503 | -7.768 | 1 | 0.030 |
| **581** | KAT7 | 760 | -7.797 | 1 | 0.035 |
| **582** | TCP1 | 453 | -7.826 | 1 | -0.042 |
| **583** | BRD2 | 599 | -7.916 | 1 | -0.051 |
| **584** | HCFC1 | 915 | -8.076 | 1 | 0.061 |
| **585** | KLF1 | 899 | -8.233 | 1 | 0.004 |
| **586** | ATF3 | 927 | -20.45 | 1 | -0.016 |
| **587** | PAX5 | 914 | -20.54 | 1 | 0.026 |
| **588** | RAG2 | 867 | -20.22 | 1 | -0.018 |
| **589** | OTX2 | 623 | -16.67 | 1 | -0.036 |
| **590** | FOS | 995 | -16.96 | 1 | -0.036 |
| **591** | GATA1 | 980 | -16.18 | 1 | -0.026 |
| **592** | NKX2-1 | 762 | -14.74 | 1 | 0.056 |
| **593** | MAFK | 1010 | -14.37 | 1 | 0.012 |
| **594** | PBX3 | 952 | -14.12 | 1 | 0.050 |
| **595** | KDM4A | 842 | -14.64 | 1 | 0.030 |
| **596** | CEBPD | 803 | -13.43 | 1 | -0.03 |
| **597** | XBP1 | 597 | -13.87 | 1 | 0.045 |
| **598** | RARA | 834 | -13.29 | 1 | 0.060 |
| **599** | MEF2B | 352 | -13.74 | 1 | -0.014 |
| **600** | MYOD1 | 510 | -12.56 | 1 | -0.035 |
| **601** | HOXA6 | 667 | -12.7 | 1 | 0.004 |
| **602** | GATA4 | 871 | -12.05 | 1 | 0.075 |
| **603** | NPAT | 530 | -12.23 | 1 | 0.061 |
| **604** | MXI1 | 986 | -12.01 | 1 | -0.026 |
| **605** | EP300 | 1022 | -12.65 | 1 | 0.055 |
| **606** | TCF21 | 290 | -11.81 | 1 | 0.049 |
| **607** | MAZ | 970 | -11.54 | 1 | 0.034 |
| **608** | TAF1 | 1005 | -11.71 | 1 | 0.006 |
| **609** | MBD4 | 480 | -11.57 | 1 | -0.003 |
| **610** | FOXP2 | 861 | -11.31 | 1 | -0.093 |
| **611** | SOX2 | 605 | -11.69 | 1 | 0.059 |
| **612** | PPARG | 839 | -10.2 | 1 | -0.101 |

| | | | | | |
|---|---|---|---|---|---|
| **613** | FOXA1 | 1017 | -10.43 | 1 | 0.042 |
| **614** | FLI1 | 923 | -10.98 | 1 | -0.022 |
| **615** | PGR | 661 | -10.97 | 1 | 0.003 |
| **616** | BHLHE40 | 951 | -10.39 | 1 | 0.081 |
| **617** | USF1 | 1002 | -10.68 | 1 | 0.086 |
| **618** | KDM1A | 618 | -10.8 | 1 | 0.062 |
| **619** | RING1 | 731 | -10.96 | 1 | -0.039 |
| **620** | ING5 | 782 | -10.48 | 1 | 0.082 |
| **621** | GATA6 | 976 | -10.56 | 1 | 0.098 |
| **622** | JARID2 | 457 | -9.195 | 1 | 0.038 |
| **623** | BARX1 | 212 | -9.714 | 1 | 0.061 |
| **624** | NCOR1 | 561 | -9.266 | 1 | -0.046 |
| **625** | HNF4A | 1009 | -9.104 | 1 | 0.050 |
| **626** | ESR1 | 978 | -9.884 | 1 | -0.019 |
| **627** | SMARCC2 | 751 | -9.853 | 1 | 0.036 |
| **628** | IKZF1 | 442 | -9.8 | 1 | -0.015 |
| **629** | PRDM11 | 297 | -9.212 | 1 | -0.083 |
| **630** | MEF2A | 716 | -9.703 | 1 | -0.038 |
| **631** | NELFA | 612 | -9.118 | 1 | -0.057 |
| **632** | ATF4 | 305 | -9.377 | 1 | 0.009 |
| **633** | KLF11 | 836 | -8.336 | 1 | -0.134 |
| **634** | POU2F2 | 926 | -8.296 | 1 | -0.02 |
| **635** | DMC1 | 386 | -8.833 | 1 | -0.034 |
| **636** | PML | 939 | -8.536 | 1 | -0.07 |
| **637** | SIX5 | 589 | -8.395 | 1 | -0.027 |
| **638** | SPI1 | 1003 | -8.825 | 1 | 0.038 |
| **639** | KMT2D | 832 | -8.434 | 1 | 0.083 |
| **640** | TLX1 | 208 | -8.992 | 1 | 0.074 |

## Supplementary Table 6 – Top 15 Regulators – Associations to melanoma

| Regulators | Adjusted p-value | Melanoma | Metastasis or tumor progression | Tumor suppressor gene | Oncogene |
|---|---|---|---|---|---|
| *FOSL2* | 4.88e-158 | | [S4.01] | | |
| *CEBPA* | 7.70e-148 | | [S4.02] | [S4.03] | |
| *ZBTB7A* | 1.76e-141 | [S4.04] | [S4.02, S4.04] | [S4.05] | |
| *SMAD1* | 1.35e-140 | [S4.06, S4.07] | [S4.08] | | |
| *GATA3* | 3.13e-136 | [S4.09] | [S4.10] | | |
| *E2F6* | 3.92e-126 | [S4.11, S4.12] | | | |
| *MITF* | 2.23e-105 | [S4.13, S4.14] | [S4.13, S4.14] | | [S4.13, S4.14] |
| *FOXP1* | 4.92e-104 | [S4.15] | [S4.16] | [S4.17] | |
| *TFAP2C* | 1.60e-96 | [S4.18] | | | |
| *RXRA* | 7.42e-94 | [S4.19, S4.20] | [S4.19, S4.20] | | |
| *CBX3* | 1.94e-89 | [S4.21] | [S4.22] | | |
| *BRCA1* | 3.03e-83 | [S4.23] | [S4.24, S4.25] | [S4.26] | |
| *ATF2* | 4.00e-78 | [S4.27] | [S4.27] | [S4.28] | [S4.28] |
| *HEY1* | 1.37e-77 | [S4.29, S4.30] | | | |
| *TP63* | 2.62e-75 | [S4.31] | [S4.32] | [S4.32] | |

[S4.01] Kim, Seung Woo, et al. "Association study of FOS-like antigen-2 promoter polymorphisms with papillary thyroid cancer in Korean population." Clinical and experimental otorhinolaryngology 7.1 (2014): 42. APA

[S4.02] Shi, Duan-Bo, et al. "C/EBPα-induced miR-100 expression suppresses tumor metastasis and growth by targeting ZBTB7A in gastric cancer." Cancer letters 369.2 (2015): 376-385.

[S4.03] Sato, Atsuyasu, et al. "CCAAT/enhancer-binding protein-α suppresses lung tumor development in mice through the p38α MAP kinase pathway." PLoS One 8.2 (2013): e57013.

[S4.04] Liu, Xue-Song, et al. "ZBTB7A suppresses melanoma metastasis by transcriptionally repressing MCAM." Molecular Cancer Research 13.8 (2015): 1206-1217.

[S4.05] Liu, Xue-Song, et al. "ZBTB7A acts as a tumor suppressor through the transcriptional repression of glycolysis." Genes & development 28.17 (2014): 1917-1928.

[S4.06] Rungsimanond, N., et al. "Stable isoform of the Smad1 protein inhibits melanoma cell proliferation and migration." JOURNAL OF INVESTIGATIVE DERMATOLOGY. Vol. 130. 75 VARICK ST, 9TH FLR, NEW YORK, NY 10013-1917 USA: NATURE PUBLISHING GROUP, 2010.

[S4.07] Tang, Ming-Rui, et al. "CSMD1 exhibits antitumor activity in A375 melanoma cells through activation of the Smad pathway." Apoptosis 17.9 (2012): 927-937.

[S4.08] Pangas, Stephanie A., et al. "Conditional deletion of Smad1 and Smad5 in somatic cells of male and female gonads leads to metastatic tumor development in mice." Molecular and cellular biology 28.1 (2008): 248-257.

[S4.09] Riker, Adam I., et al. "The gene expression profiles of primary and metastatic melanoma yields a transition point of tumor progression and metastasis." BMC medical genomics 1.1 (2008): 13.

[S4.10] Chou, Jonathan, et al. "GATA3 suppresses metastasis and modulates the tumour microenvironment by regulating microRNA-29b expression." Nature cell biology 15.2 (2013): 201-213.

[S4.11] Deng, Tao, et al. "An essential role for DNA methyltransferase 3a in melanoma tumorigenesis." Biochemical and biophysical research communications 387.3 (2009): 611-616.

[S4.12] Halaban, Ruth, et al. "Deregulated E2F transcriptional activity in autonomously growing melanoma cells." The Journal of experimental medicine 191.6 (2000): 1005-1016. APA

[S4.13] Levy, Carmit, Mehdi Khaled, and David E. Fisher. "MITF: master regulator of melanocyte development and melanoma oncogene." Trends in molecular medicine 12.9 (2006): 406-414.

[S4.14] Garraway, Levi A., et al. "Integrative genomic analyses identify MITF as a lineage survival oncogene amplified in malignant melanoma." Nature 436.7047 (2005): 117-122.

[S4.15] Spritz, Richard A. "The genetics of generalized vitiligo: autoimmune pathways and an inverse relationship with malignant melanoma." Genome medicine 2.10 (2010): 78.

[S4.16] Feng, Jian, et al. "High expression of FoxP1 is associated with improved survival in patients with non–small cell lung cancer." American journal of clinical pathology 138.2 (2012): 230-235.

[S4.17] Banham, Alison H., et al. "The FOXP1 winged helix transcription factor is a novel candidate tumor suppressor gene on chromosome 3p." Cancer research 61.24 (2001): 8820-8829.

[S4.18] Penna, Elisa, et al. "microRNA-214 contributes to melanoma tumour progression through suppression of TFAP2C." The EMBO journal 30.10 (2011): 1990-2007.

[S4.19] Chakravarti, Nitin, et al. "Decreased expression of retinoid receptors in melanoma: entailment in tumorigenesis and prognosis." Clinical Cancer Research 13.16 (2007): 4817-4824.

[S4.20] Klopper, Joshua P., et al. "Combination PPAR and RXR Agonist Treatment in Melanoma Cells: Functional Importance of S100A2." PPAR research 2010 (2009).

[S4.21] Schmid, Rainer, et al. "Melanoma inhibitory activity promotes melanoma development through activation of YBX1." Pigment cell & melanoma research 26.5 (2013): 685-696.

[S4.22] Hammoudi, Abeer T. Proteomic dissection of the early genetic changes in a colorectal cancer model. Diss. University of Liverpool, 2012.

[S4.23] Liede, Alexander, Beth Y. Karlan, and Steven A. Narod. "Cancer risks for male carriers of germline mutations in BRCA1 or BRCA2: a review of the literature." Journal of Clinical Oncology 22.4 (2004): 735-742.

[S4.24] Seery, Liam T., et al. "BRCA1 expression levels predict distant metastasis of sporadic breast cancers." International journal of cancer 84.3 (1999): 258-262.

[S4.25] Szabova, Ludmila, et al. "Perturbation of Rb, p53, and Brca1 or Brca2 cooperate in inducing metastatic serous epithelial ovarian cancer." Cancer research 72.16 (2012): 4141-4153.

[S4.26] Chang, Suhwan, et al. "Tumor suppressor BRCA1 epigenetically controls oncogenic microRNA-155." Nature medicine 17.10 (2011): 1275-1282.

[S4.27] Bhoumik, Anindita, et al. "An ATF2-derived peptide sensitizes melanomas to apoptosis and inhibits their growth and metastasis." The Journal of clinical investigation 110.5 (2002): 643-650.

[S4.28] Bhoumik, Anindita, and Ze'ev Ronai. "ATF2: a transcription factor that elicits oncogenic or tumor suppressor activities." Cell cycle 7.15 (2008): 2341-2345.

[S4.29] Bedogni, Barbara, et al. "Notch1 is an effector of Akt and hypoxia in melanoma development." The Journal of clinical investigation 118.11 (2008): 3660-3670.

[S4.30] Hoek, Keith, et al. "Expression profiling reveals novel pathways in the transformation of melanocytes to melanomas." Cancer research 64.15 (2004): 5270-5282.

[S4.31] Matin, Rubeta N., et al. "p63 is an alternative p53 repressor in melanoma that confers chemoresistance and a poor prognosis." Journal of Experimental Medicine (2013): jem-20121439.

[S4.32] Su, Xiaohua, Deepavali Chakravarti, and Elsa R. Flores. "p63 steps into the limelight: crucial roles in the suppression of tumorigenesis and metastasis." Nature Reviews Cancer 13.2 (2013): 136-143.

## Supplementary Table 7 – Macrophages

| TF | Regression Coefficient | |
|---|---|---|
| **HOXA3** | 0.148367202 | [S6.01] |
| **HLTF** | 0.089891363 | [S6.02] |
| **ETV5** | 0.078639561 | [S6.03] |
| **GMEB1** | 0.045548794 | [S6.04] |
| **HOXA5** | 0.04494131 | [S6.05] |
| **NRF1** | 0.039230003 | [S6.06, S6.07, S6.08] |
| **PAX2 (paralog of PAX5)** | 0.038496525 | [S6.09] |
| **ETS2** | 0.034375522 | [S6.10] |
| **ELF5** | 0.033069267 | [S6.11] |
| **NFATC1** | 0.032528012 | [S6.12] |
| **KLF4** | 0.025539592 | [S6.13] |
| **NKX2.5** | 0.025418796 | [S6.14] |
| **RAD21** | -0.052709626 | [S6.15] |

[S6.01] Al Sadoun, Hadeel, et al. "Enforced Expression of Hoxa3 Inhibits Classical and Promotes Alternative Activation of Macrophages In Vitro and In Vivo." The Journal of Immunology 197.3 (2016): 872-884.

[S6.02] Lahouassa, Hichem, et al. "HIV-1 Vpr degrades the HLTF DNA translocase in T cells and macrophages." Proceedings of the National Academy of Sciences 113.19 (2016): 5311-5316.

[S6.03] Roy, Sugata, et al. "Redefining the transcriptional regulatory dynamics of classically and alternatively activated macrophages by deepCAGE transcriptomics." Nucleic acids research 43.14 (2015): 6969-6982.

[S6.04] Kawabe, Kiyokazu, et al. "IL-12 inhibits glucocorticoid-induced T cell apoptosis by inducing GMEB1 and activating PI3K/Akt pathway." Immunobiology 217.1 (2012): 118-123.

[S6.05] Gordon, Siamon, ed. The macrophage as therapeutic target. Vol. 158. Page 14, Springer Science & Business Media, ISBN 978-3-642-55742-2, 2012.

[S6.06] Beyer, T. A., et al. "Roles and mechanisms of action of the Nrf2 transcription factor in skin morphogenesis, wound repair and skin cancer." Cell death and differentiation 14.7 (2007): 1250.

[S6.07] Liou, Geou-Yarh, and Peter Storz. "Reactive oxygen species in cancer."

[S6.08] Zhang, Shu-Tao, et al. "Nrf1 is time-dependently expressed and distributed in the distinct cell types after trauma to skeletal muscles in rats." Histol Histopathol 28 (2013): 725-735. Free radical research 44.5 (2010): 479-496.

[S6.09] Hodawadekar, Suchita, et al. "B-lymphoma cells with epigenetic silencing of Pax5 trans-differentiate into macrophages, but not other hematopoietic lineages." Experimental cell research 313.2 (2007): 331-340.

[S6.10] Zabuawala, Tahera, et al. "An ets2-driven transcriptional program in tumor-associated macrophages promotes tumor metastasis." Cancer research 70.4 (2010): 1323-1333.

[S6.11]Tuomisto, Tiina T., et al. "Analysis of gene and protein expression during monocyte-macrophage differentiation and cholesterol loading—cDNA and protein array study." Atherosclerosis 180.2 (2005): 283-291.

[S6.12] Zanoni, Ivan, and Francesca Granucci. "Regulation and dysregulation of innate immunity by NFAT signaling downstream of pattern recognition receptors (PRRs)." European journal of immunology 42.8 (2012): 1924-1931.

[S6.13] Liao, Xudong, et al. "Krüppel-like factor 4 regulates macrophage polarization." The Journal of clinical investigation 121.7 (2011): 2736-2749. Schuetz, Anja, et al. "The structure of the Klf4 DNA-binding domain links to self-renewal and macrophage differentiation." Cellular and Molecular Life Sciences 68.18 (2011): 3121-3131.

[S6.14] Ravasi, Timothy, et al. "Generation of diversity in the innate immune system: macrophage heterogeneity arises from gene-autonomous transcriptional probability of individual inducible genes." The Journal of Immunology 168.1 (2002): 44-50.

[S6.15] Bowers, Sarion R., et al. "A conserved insulator that recruits CTCF and cohesin exists between the closely related but divergently regulated interleukin-3 and granulocyte-macrophage colony-stimulating factor genes." Molecular and cellular biology 29.7 (2009): 1682-1693.
